# KDM6 Demethylases Contribute to EWSR1::FLI1-Driven Oncogenic Reprogramming in Ewing Sarcoma

**DOI:** 10.1158/0008-5472.CAN-24-3452

**Published:** 2025-10-14

**Authors:** Elisabet Figuerola-Bou, Carla Ríos-Astorch, Enrique Blanco, María Sánchez-Jiménez, Pablo Táboas, Guerau Fernández, Soledad Gómez-González, Oscar Muñoz, Pol Castellano-Escuder, Sara Perez-Jaume, Marta Garcia, Estela Prada, Silvia Mateo-Lozano, Nicolo Riggi, Alexandra Avgustinova, Cinzia Lavarino, Luciano Di Croce, Sara Sánchez-Molina, Jaume Mora

**Affiliations:** 1 Pediatric Cancer, Institut de Recerca Sant Joan de Déu (IRSJD), Barcelona, Spain.; 2 Center for Genomic Regulation (CRG), Barcelona Institute of Science and Technology, Barcelona, Spain.; 3 Universitat Pompeu Fabra (UPF), Barcelona, Spain.; 4 Molecular Genetics Department, IRSJD, Barcelona, Spain.; 5 Biomarkers and Nutritional & Food Metabolomics Research Group, Department of Nutrition, Food Science and Gastronomy, University of Barcelona, Barcelona, Spain.; 6 Biostatistics Unit, Department of Clinical Practice, University of Barcelona, Barcelona, Spain.; 7 Faculty of Biology and Medicine, Institute of Pathology, Centre Hospitalier Universitaire Vaudois, University of Lausanne, Lausanne, Switzerland.; 8 Department of Cell and Tissue Genomics, Genentech Inc., South San Francisco, California.; 9 Institute for Research in Biomedicine (IRB Barcelona), Barcelona Institute of Science and Technology, Barcelona, Spain.; 10 Pediatric Cancer Center Barcelona (PCCB), Hospital Sant Joan de Déu (HSJD), Barcelona, Spain.; 11 Institució Catalana de Recerca i Estudis Avançats (ICREA), Barcelona, Spain.

## Abstract

Ewing sarcoma is a highly aggressive tumor arising in bones and soft tissues, driven by the fusion oncoprotein EWSR1::FLI1. This aberrant transcription factor binds to GGAA microsatellites, causing epigenetic reprogramming through the formation of active neoenhancers in a permissive cellular context. Inhibition of the oncogene remains challenging, and current efforts seek to exploit emergent epigenetic treatments targeting EWSR1::FLI1 cofactors. In Ewing sarcoma, neoenhancers are characterized by strong enrichment of the active H3K27ac mark and a concomitant lack of the repressive H3K27me3 mark. These regions are typically decorated with high levels of H3K27me3 prior to EWSR1::FLI1 activation. In this study, upon expression of EWSR1::FLI1 in human pediatric mesenchymal stem cells, considered the putative cell of origin of Ewing sarcoma, we unraveled the genome-wide redistribution of H3K27me3. Stemming from these results, we elucidated the contribution of the H3K27me3 demethylases KDM6A/UTX and KDM6B/JMJD3 in the transcriptional activity of EWSR1::FLI1-induced enhancers. KDM6A had a demethylase-independent role in recruiting BRG1 at EWSR1::FLI1-primed enhancers containing single GGAA motifs, which was critical for Ewing sarcoma tumor growth. Conversely, KDM6B demethylated H3K27me3 at specific EWSR1::FLI1-active enhancers, colocalizing with BRG1 at GGAA repeats. Loss of KDM6B impaired the growth of Ewing sarcoma tumor xenografts. These results highlight KDM6 demethylases as EWSR1::FLI1 functional partners with potential as targets for treating Ewing sarcoma.

**Significance:**

KDM6A and KDM6B mediate critical mechanisms behind EWSR1::FLI1 transcriptional activation program involving demethylase-independent and dependent functions, respectively, supporting the development of therapeutic strategies targeting these demethylases in Ewing sarcoma.

## Graphical Abstract

**Figure:**
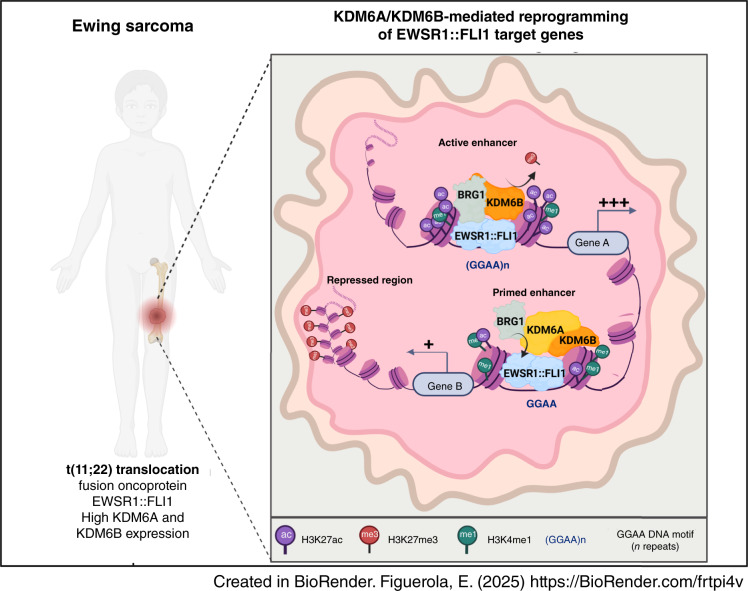

## Introduction

Ewing sarcoma is an aggressive neoplasia arising in the bones and soft tissues of children, adolescents, and young adults (1). Whole-genome sequencing studies of Ewing sarcoma tumors reported remarkably stable genomes with a very low mutational burden (2, 3). Like many developmental cancers, Ewing sarcoma is characterized by a differentiation block during development conferred by the expression of an oncogenic driver (4). The characteristic reciprocal chromosomal translocation involves, most commonly (85% of cases), the *EWS RNA Binding Protein 1* (*EWSR1*) gene on chromosome 22 and the ETS family member *Friend Leukemia Integration 1 Transcription Factor* (*FLI1*) on chromosome 11 (5). The resulting fusion protein, EWSR1::FLI1, retains the transactivation domain of *EWSR1* fused to the DNA-binding domain of *FLI1* and acts as an aberrant transcription factor (6, 7). *EWSR1* can be fused to other members of the ETS gene family, such as the *ETS-Related Gene* (*ERG*), producing the *EWSR1::ERG* translocation (10% of cases). Noticeably, EWSR1::FLI1 can only achieve oncogenic transformation in the right cellular background (8–10). Although EWSR1::FLI1 induces growth arrest or apoptosis in differentiated primary cells (11, 12), its expression in human mesenchymal stem cells (hMSC) recapitulates the Ewing sarcoma gene signature, with stronger induction of oncogenic targets in human pediatric MSC (hpMSC; refs. 9, 13).

EWSR1::FLI1 exhibits a high affinity for binding DNA through GGAA motifs, a class of ETS-specific response elements, activating or repressing their targets based on the number of motif repeats (14, 15). At GGAA repeats, multimers of EWSR1::FLI1 establish *de novo* active enhancers (neoenhancers) by promoting chromatin opening through the recruitment of chromatin-modifying complexes. Indeed, in Ewing sarcoma cell lines and primary tumors, the vast majority of EWSR1::FLI1 binding sites are decorated by H3K27ac, a posttranslational histone modification associated with active enhancers (16, 17). Consistently, EWSR1::FLI1 features scaffolding properties and, at GGAA repeats, recruits core members of the mammalian BRG1/BRM-Associated Factor (BAF) complex, such as BAF155, that are critical for transcriptional activation (18). Furthermore, we have described that RING1B, a member of the Polycomb group family of proteins (19), facilitates EWSR1::FLI1 recruitment toward key enhancers (20). Although the mechanism behind EWSR1::FLI1 gene repression is less well understood, it has been proposed that the binding of EWSR1::FLI1 monomers on single instances of the GGAA motifs causes the displacement of endogenous ETS transcription factors, decreasing transcriptional activation (16).

H3K27me3 mark restricts cell fate by limiting chromatin accessibility to key developmental genes (21). EZH2, the core member of the Polycomb Repressive Complex 2 (PRC2), is the primary enzymatic writer of H3K27me3 (22, 23). Removal of H3K27me3, concerted with promoter activation, is mediated by members of the KDM6 family of demethylases, including KDM6A (UTX) and KDM6B (JMJD3), through their JmjC domain (24, 25) and determines the specification of human neural progenitor cells (26). KDM6A has been described as a partner of the SET1/MLL complex, which is responsible for gene expression by methylation of H3K4 at active enhancers (27, 28), whereas KDM6B has been reported to cooperate with the transcription factor KLF4 in enhancer-driven reprogramming and with SMAD3 in neural-specific enhancers (29, 30).

Deregulation of the H3K27me3 balance that is governed by the coordinated enzymatic activities of EZH2 and KDM6A/KDM6B leads to differentiation defects and cancer (31). EZH2 has been involved in the tumor progression mechanisms of a variety of cancer types (32), whereas KDM6A and KDM6B have been identified in numerous malignancies with either oncogenic or tumor suppressor roles (33–37). Indeed, EZH2 is a well-known directly activated target of EWSR1::FLI1 that blocks neuroectodermal and endothelial differentiation in Ewing sarcoma (9, 38). We previously demonstrated that EWSR1::FLI1 occupies weakly repressed Polycomb chromatin states in hMSC (20). Although EWSR1::FLI1-bound GGAA repeats are devoid of H3K27me3 both in Ewing sarcoma cell lines and primary tumors (16, 17), in human umbilical vein endothelial cells and hpMSC, these regions are extensively decorated with H3K27me3 (8, 39). However, the precise mechanisms behind the equilibrium of H3K27me3 deposition and erasure, as well as its impact on Ewing sarcoma tumor development, have not yet been addressed.

In this study, we shed light on the dynamics of H3K27me3 removal by KDM6A and KDM6B demethylases within the context of Ewing sarcoma. First, we show how H3K27me3 is redistributed genome-wide during Ewing sarcoma tumorigenesis in hpMSCs. Next, we demonstrate that KDM6A and KDM6B bind to the same genomic regions as EWSR1::FLI1, with KDM6A decorating EWSR1::FLI1-primed enhancers containing GGAA single motifs and KDM6B characteristically enriched at active enhancers containing multimeric GGAA repeats. Importantly, both KDM6A and KDM6B demethylases are involved in EWSR1::FLI1-related transcriptional activation in a demethylase-independent and demethylase-dependent manner, respectively, being critical for Ewing sarcoma tumor growth. Our findings provide deeper knowledge about the specific functions of H3K27me3 KDM6 demethylases in Ewing sarcoma and support the development of epigenetic strategies based on the reversibility of the processes involved in the H3K27me3 equilibrium.

## Materials and Methods

### hpMSCs and cell lines

hpMSCs were extracted from the healthy bone marrow of pediatric donors from Hospital Sant Joan de Déu (Barcelona, Spain; HSJD) and characterized according to described protocols (9, 40, 41). Cells were cultured at low confluence with Iscove’s modified Dulbecco’s medium supplemented with 10% fetal newborn calf serum, 1% penicillin/streptomycin (Gibco), and 10 ng/mL of PDGF-BB (PeproTech).

The Ewing sarcoma cell lines, A-673 (RRID:CVCL_0080), TC-71 (RRID:CVCL_2213), and RM-82 (RRID:CVCL_9714), were purchased from the ATCC. A-673/TR/shEF cells, which harbor a doxycycline-inducible short hairpin RNA (shRNA) targeting EWSR1::FLI1 mRNA, have been previously characterized (42). All cell lines were authenticated by short tandem repeat profiling and routinely screened for *Mycoplasma* and were found to be negative throughout the study. They were cultured in RPMI 1640 media and supplemented with 10% FBS, L-glutamine, and penicillin/streptomycin (Gibco). Cells were maintained in a humidified chamber at 37°C and 5% CO_2_ and split every 2 to 3 days when reaching confluence. Ewing sarcoma cells were treated with the KDM6A/KDM6B demethylase inhibitor GSKJ4 (Selleckchem) at 2.5 and 5 μmol/L for 72 hours. DMSO (Sigma-Aldrich) was used as a vehicle control. To induce EWSR1::FLI1 knockdown, A-673/TR/shEF cells were treated with doxycycline (1 μg/mL) for 72 hours (Sigma-Aldrich).

### Patient samples

Biopsies from 45 primary Ewing sarcoma tumors at diagnosis from the HSJD Biobank, integrated into the Spanish Biobank Network of ISCIII and Xarxa de Tumors de Catalunya, were used for experimental purposes in agreement with the ethical committee procedures. Two samples were not appraisable for technical reasons and were excluded. Studies were conducted in accordance with the Declaration of Helsinki and were approved by the Institutional Review Boards at Fundació Sant Joan de Déu and HSJD. Written informed consent was obtained from patients or their legal guardians before the collection of samples.

### Lentiviral and transient transfections

Infection was performed as previously described (20). In both overexpression and knockdown experiments, infected cells were selected with 0.5 to 1 μg/mL puromycin (Sigma-Aldrich) for 72 hours and maintained in the first passages. Lentiviral SMARTvector inducible Tet-On shRNA plasmids (GE Healthcare Dharmacon) and sequences targeting KDM6A or KDM6B are described in Supplementary Table S1. Induction of the shRNA was performed with doxycycline hyclate (Sigma-Aldrich) at 2 μg/mL for 72 hours. The empty and EWSR1::FLI1-pLIV vector was kindly provided by Dr. Rivera (18). For dead mutant experiments, A-673 KDM6A KO cells were infected with the pLV[Exp]-Puro-EF1A vector expressing either wild type (WT) or the catalytically inactive form with H1146A and E1148A mutations of KDM6A (43). Both nontargeting control (sgCTRL) and KDM6A KO cells were infected with the empty pLV[Exp]-Puro-EF1A vector as controls. Validation of the overexpression or knockdown was determined by Western blot.

Ewing sarcoma cell lines were seeded in six-well plates at 0.25 × 10^6^ cells/well and transfected with Lipofectamine RNAiMAX (Invitrogen) using 30 pmol of siRNA in Opti-MEM media (Gibco) following the manufacturer’s instructions. siRNA sequences are described in Supplementary Table S1.

### CRISPR-Cas9 genome editing

KDM6A and KDM6B knockout (KO) cells were generated using the Gene Knockout Kit version 2 (Synthego), containing a pool of three validated single-guide (sg) RNAs (sgRNA) and exogenous Cas9, following the manufacturer’s guidelines. CRISPR-edited cell pools were isolated by limit dilution in 96-well plates. Isolated clones were expanded, and cells were collected for subsequent validation of the PCR product by Sanger sequencing. The Inference of CRISPR Edits (ICE) analysis software (RRID:SCR_024508) was used to analyze the obtained sequences of edited cells (clones with a model fit R^2^ > 0.6 were only considered). KO clones were further validated by Western blot. Target sequences for the pool of three commercially predesigned sgRNAs targeting KDM6A or KDM6B (Synthego) are described in Supplementary Table S1.

### Clonogenic assays

Clonogenic assays were performed by seeding 0.12 × 10^4^ cells/well in six-well plates and changing the media every 2 to 3 days until visible colonies were grown. Cells were then fixed for 10 minutes with 4% paraformaldehyde (Sigma-Aldrich), washed with PBS, and incubated with a crystal violet solution (2% W/V, 20% methanol in PBS; Sigma-Aldrich) for 5 minutes. Quantification of the colony number was performed with ImageJ (RRID:SCR_003070) plug-in ColonyArea (44).

### RNA extraction and RT-qPCR

Total RNA was isolated and purified using the RNeasy Mini Kit (QIAGEN) following the manufacturer’s instructions. Quantification of RNA samples was performed using a NanoDrop 1000 spectrophotometer (RRID:SCR_016517). Reverse transcription (RT) was performed using 1 μg of purified RNA and converted to cDNA with the retrotranscriptase M-MLV Reverse Transcriptase, the RNasin Plus RNase inhibitor, and random primers (Promega). SYBR Green PCR Master Mix (Thermo Fisher Scientific) was used to perform qPCR with the QuantStudio 6 Flex (RRID:SCR_020239) using specific primer sequences (see Supplementary Table S1). The obtained data were normalized to a housekeeping gene and analyzed with the 2^−ΔΔCT^ method relative to the experimental control condition. Data were obtained from at least three independent biological experiments and expressed as mean ± SEM.

### RNA sequencing and functional analysis

RNA sequencing (RNA-seq) libraries were prepared with 0.5 to 1 μg of total high-quality RNA collected from samples and the Illumina Stranded Total RNA Prep kit (Illumina) according to the manufacturer’s instructions. Fastq files were analyzed using FastQC software (RRID:SCR_014583) to assess read quality. Adapters were removed, and reads were trimmed using Cutadapt software (RRID:SCR_011841) according to per-base Phred quality scores and minimum length. Reads were pseudoaligned to GRCh37 using Kallisto (RRID:SCR_016582). Gene read counts were used to determine differential gene expression using R packages tximport (RRID:SCR_016752) and DESeq2 (RRID:SCR_015687). ERCC spike-ins (Thermo Fisher Scientific) were used for sample normalization, and batch effects were removed using LIMMA (RRID:SCR_010943). Heatmaps were generated with the R package pheatmap (RRID:SCR_016418). Gene set enrichment analysis (RRID:SCR_003199) was used to determine biologically relevant transcriptional events in gene sets from Hallmark Collection running each list with the knockdown. Reports of functional enrichments of Gene Ontology (GO) and other genomic libraries were generated using Enrichr (RRID:SCR_001575). Additionally, publicly available RNA-seq data from A-673 (16) and EWSR1::ETS-knockdown Ewing sarcoma cell lines (45) were also included in the analysis.

### IHC

Analyses were performed following standard protocols. Fixed tumor xenografts were embedded in paraffin and consecutively cut into 2 μm thick slices. Sections of paraffin tumors were deparaffinized, rehydrated in an alcohol battery, and incubated with antigen retrieval with epitope retrieval solution (pH 6.0; Novocastra Laboratories). Blocking with endogenous peroxidase was performed with Protein Block (Novocastra Laboratories) for 5 minutes, and subsequent steps were performed in the DAKO Autostainer Link 48 (Agilent). Slides were counterstained in hematoxylin and eosin, dehydrated with alcohol and xylene, and finally cover-slipped with DPX mounting medium (Sigma-Aldrich). NanoZoomer 2.0 (RRID:SCR_021658) was used to scan selected tumors for digital image processing. For KDM6A and KDM6B stains in Ewing sarcoma tumors, IHC semiquantification was scored by an independent pathologist. A semiquantitative histoscore (*H*-score) value was calculated based on a linear combination of the intensity and proportion of stained cells per camp that punctuates the percentage of strongly stained nuclei (SSN), moderately stained nuclei (MSN), and weakly stained nuclei (WSN) following the formula: *H*-score = 1 × WSN + 2 × MSN + 3 × SSN. The *H*-score value ranges from possible scores of 0 to 300 (46). For tumor xenografts, the *H*-score was calculated with IHC Profiler (RRID:SCR_023577) and ImageJ software based on staining intensity and the percentage of positive staining cells. The primary antibodies and dilutions used are listed in Supplementary Table S1.

### Protein extract preparation and Western blotting

Whole cell protein extracts were prepared in RIPA buffer [10 mmol/L Tris-HCl (pH 8), 1 mmol/L EDTA, 0.5 mmol/L EGTA, 1% Triton X-100, 0.1% sodium deoxycholate, 0.1% SDS, and 140 mmol/L NaCl] containing phosphatase and EDTA-free Protease Inhibitor Cocktail (Sigma-Aldrich). Cell lysates incubated on ice for 30 minutes were centrifuged at 12,000 rpm for 15 minutes at 4°C. Histone extracts were isolated using the EpiQuik Histone Extraction Kit (Abcam) following the manufacturer’s instructions. Protein supernatants were collected and quantified by Bradford assay. Fifty micrograms of whole cell extract or 5 μg of histone protein extracts were mixed with loading Laemmli buffer with DTT for Western blot experiments following standard protocols. The primary antibodies and dilutions are listed (see Supplementary Table S1), and the secondary antibodies, goat anti-rabbit and goat anti-mouse IRDye (RRID:AB_2651128, RRID:AB_2721181; Li-COR), were diluted 1:10,000 and incubated for 1 hour at room temperature to blot membranes. Nitrocellulose membranes (Sigma-Aldrich) were scanned and visualized with the Li-COR Odyssey Infrared Imaging System (RRID:SCR_014579).

### Chromatin immunoprecipitation–qPCR

Chromatin immunoprecipitation (ChIP)–qPCR assays were performed as previously described (47). Cultured cells were fixed using 1% methanol-free formaldehyde (Thermo Fisher Scientific) for 10 minutes at room temperature, and cross-linking was stopped by adding 500 μL of glycine (1.25 mol/L). Lysis was performed in soft lysis buffer (0.1% SDS, 0.15 mol/L NaCl, 1% Triton X-100, 1 mmol/L EDTA, and 20 mmol/L Tris pH 8) supplemented with 1 mg/mL of protease inhibitors (Sigma-Aldrich). Cell lysates were sonicated for four cycles of 10 minutes (30-second intervals) with a Bioruptor Pico (RRID:SCR_023470) until chromatin was sheared to an average length of 200 bp. After centrifugation, a small fraction of eluted chromatin was measured with a Qubit fluorometer (RRID:SCR_020553). Immunoprecipitations were prepared starting with 30 μg of chromatin for each antibody and incubated overnight at 4°C in a rotating wheel (see Supplementary Table S1). Fifty microliters of Dynabeads Protein A (Invitrogen) were added to the samples, and the slurry was incubated for 2 hours to capture DNA fragments. Immunoprecipitates were washed with the following buffers: TSE I [0.1% SDS, 1% Triton X-100, 2 mmol/L EDTA, 20 mmol/L Tris–HCl (pH 8), 150 mmol/L NaCl], TSE II [0.1% SDS, 1% Triton X-100, 2 mmol/L EDTA, 20 mmol/L Tris–HCl (pH 8), 500 mmol/L NaCl], TSE III [0.25 mol/L LiCl, 1% Nonidet P-40, 1% deoxycholate, 1 mmol/L EDTA, 10 mmol/L Tris–HCl (pH 8)], and Tris-EDTA buffer. All incubation and washing steps were performed in a rotating wheel at 4°C to avoid protein degradation. DNA captured by the beads was eluted by adding 120 μL of a solution containing 1% SDS, 0.1 mol/L NaHCO_3_ and de-cross-linked at 65°C for 3 hours with gentle shaking. Genomic DNA fragments from ChIP samples were purified with the QIAquick PCR Purification Kit (QIAGEN) and eluted in 50 to 100 μL of Tris-EDTA buffer. Differences in DNA content from ChIP assays were determined by qPCR using the SQ6 Real-Time PCR System (RRID:SCR_020239) and SYBR Green master mix (Thermo Fisher Scientific). The reported data from at least three independent experiments represent qPCR values normalized to input DNA and are expressed as a percentage of bound/input signal and presented as mean ± SEM.

### ChIP sequencing and bioinformatic analysis

ChIP sequencing (ChIP-seq) libraries were prepared using 2 to 5 ng of input and ChIP samples and the NEBNext Ultra DNA Library Prep Kit for Illumina (New England Biolabs) following the manufacturer’s protocol. All purification steps were performed using Agencourt AMPure XP beads (QIAGEN). NEBNext multiplex oligonucleotides for Illumina (New England Biolabs) were used for library amplification. Quality control and fragment size were analyzed using Agilent High Sensitivity ChIP and quantified with the KAPA Library Quantification Kit (Roche). ChIP-seq data from DNA and input samples were sequenced with the HiSeq 2500 Illumina sequencing system (RRID:SCR_016383).

ChIP-seq samples were mapped against the hg19 human genome assembly using Bowtie (RRID:SCR_005476) with the option -m 1 to discard those reads that could not be uniquely mapped to just one region. ChIP-seq samples normalized by 1% spike-in were mapped against a synthetic genome constituted by the human and fruit fly chromosomes (hg19 + dm3) using Bowtie (RRID:SCR_005476) with the option -m 1 to discard reads that did not map uniquely to one region. MACS (RRID:SCR_013291) was run with the default parameters but with the shift size adjusted to 100 bp to perform the peak calling against the corresponding control sample. DiffBind (RRID:SCR_012918) was run next over the union of peaks from each pair of replicates of the same experiment to find the peaks significantly enriched in both replicates in comparison with the corresponding controls (DiffBind 2.0 arguments: categories = DBA_CONDITION, block = DBA_REPLICATE, and method = DBA_DESEQ2_BLOCK).

We used SeqCode (RRID:SCR_018070) for ChIP-seq downstream analysis across multiple stages: (i) The genome distribution of each set of peaks was generated by counting the number of peaks fitted on each class of region according to RefSeq (RRID:SCR_003496) annotations. The promoter is the region between 2.5 kb upstream and 2.5 kb downstream of the transcription start site (TSS). Genic regions correspond to the rest of the gene (the part that is not classified as a promoter), and the rest of the genome is considered to be intergenic. Peaks that overlapped with more than one genomic feature were proportionally counted the same number of times; (ii) aggregated plots showing the average distribution of ChIP-seq reads around the TSS or along the gene body of each target gene were generated by counting the number of reads for each region according to RefSeq and then averaging the values for the total number of mapped reads of each sample and the total number of genes in the particular gene set; (iii) heatmaps displaying ChIP-seq signal strength around the summit of each peak were generated by counting the number of reads in this region for each individual peak and normalizing this value with the total number of mapped reads of the sample. Peaks on each heatmap were ranked by the logarithm of the average number of reads in the same genomic region; (iv) box plots showing the ChIP-seq level distribution for a particular ChIP experiment on a set of genomic peaks were calculated by determining the maximum value in this region for this sample, which was assigned afterward to the corresponding peak. To quantify genome-wide differences in H3K27me3 gain/loss, we performed bin mapping analysis with a size of 1 Kbp as this is the average size for H3K27me3 peaks (average value per bin, genome assembly: hg19); (v) bedGraph profiles were generated from each set of mapped reads and uploaded into the UCSC Genome Browser (RRID:SCR_005780); and (vi) the set of target genes of a biological feature was found by matching all ChIP-seq peaks in the region 2.5 kb upstream of the TSS to the end of the transcripts as annotated in RefSeq.

To build our collection of enhancers and promoters, we reanalyzed published ChIP-seq samples of H3K4me1, H3K27ac, H3K27me3, and H3K4me3 in A-673 cells (16) as in ref. 20. We defined five classes of regulatory elements: active enhancers (H3K27ac), active promoters (H3K27ac + H3K4me3), poised enhancers (H3K27me3), primed enhancers (H3K4me1 without H3K27ac), and bivalent promoters (H3K27me3 + H3K4me3). To construct the list of putative targets of KDM6A/KDM6B enhancers, we identified the genes in the vicinity of overlapping EWSR1::FLI1-KDM6A and EWSR1::FLI1-KDM6B modules (maximum distance between peaks and differentially regulated genes: 100 kb). Reports of functional enrichments of GO and other genomic libraries were generated using Enrichr (RRID:SCR_001575). Motif analysis of the sequences of ChIP-seq peaks was performed with the MEME Suite–motif-based sequence analysis tools (RRID:SCR_001783), adjusting the MEME motif width between 5 and 15 bp. ChIP-seq raw data from control and EWSR1::FLI1 hpMSC were kindly provided by Dr. Miguel Rivera. For ChIP-seq experiments with EWSR1::FLI1 in Ewing sarcoma cell lines, we used an antibody that correlates 62% at peak level with published data (16) and nicely reproduces our previous data with RING1B overlap (20).

### Murine xenograft studies

*In vivo* studies were performed after the approval of the Institutional Animal Research Ethics Committee and the animal experimentation commission of the Catalan government. Cells (1 × 10^6^) of parental, KDM6 KO (sgRNA#1 and sgRNA#2), and sgCTRL A-673 cells in 200 µL of PBS with Matrigel (Becton Dickinson) were subcutaneously injected into two flanks of athymic nude mice (RRID:IMSR_ENV:HSD-069; *n* = 11 for parental, *n* = 9 for sgCTRL, and *n* = 11 for sgKDM6A#1; *n* = 9 for parental, *n* = 12 for sgCTRL, and *n* = 7 for sgKDM6A#2; *n* = 8 for sgCTRL; and *n* = 11 for sgKDM6B#1 and sgKDM6B#2; *n* indicates the number of tumors). The same procedure was performed for the *in vivo* experiment of the reintroduction of a WT and a dead mutant enzyme form (H1146A/E1148A) of KDM6A (sgKDM6A WT and sgKDM6A-mut, respectively) in A-673 cells (*n* = 11 for sgCTRL and sgKDM6A#1 + KDM6A-mut, *n* = 9 for sgKDM6A#1, and *n* = 8 for sgKDM6A#1 + KDM6A WT). Tumor growth was monitored 3 times per week by measuring growing tumors with a digital caliper. Mice were sacrificed when tumors reached a volume of 1,500 mm^3^, and tumors were excised. Collected tumors were divided into parts. One part was frozen for protein experiments, and the other was fixed in 10% formalin for IHC experiments. For RNA experiments, four tumors from each experimental group were dissociated with collagenase IV (50 mg/mL; Sigma-Aldrich) and a tissue chopper (RRID:SCR_015798). Then tissue homogenates were digested with 5 mg/mL of DNase I and 0.25% trypsin/EDTA (Sigma-Aldrich) to subsequently separate mouse stromal cells from human cells using a Mouse Cell Depletion kit (Miltenyi Biotec) following the manufacturer’s guidelines. The log-rank test was used to calculate the significance of the groups in Kaplan–Meier.

### Quantification and statistical analyses

Data were analyzed using GraphPad Prism (RRID:SCR_002798) version 9.1.2 and expressed as mean ± SEM or SD as indicated in figure legends. Kruskal–Wallis one-way ANOVA and two-way ANOVA with Tukey correction (for non-normally distributed data) were applied to determine differences between multiple groups. Holm–Šídák or Dunn tests were used for multiple comparison tests. Student *t* test and Mann–Whitney *t* test (for non-normally distributed data) were used for nonpaired comparisons of two groups. Kaplan–Meier curves were compared with the log-rank (Mantel–Cox) test. Statistically significant differences among groups are annotated in each graph of the article, and the statistical tests applied can be found in the figure legends. *, *P* < 0.05; **, *P* < 0.01; ***, *P* < 0.001; ****, *P* < 0.0001.

## Results

### H3K27me3 genome-wide redistribution upon EWSR1::FLI1 overexpression in hpMSCs

**Figure 1.**
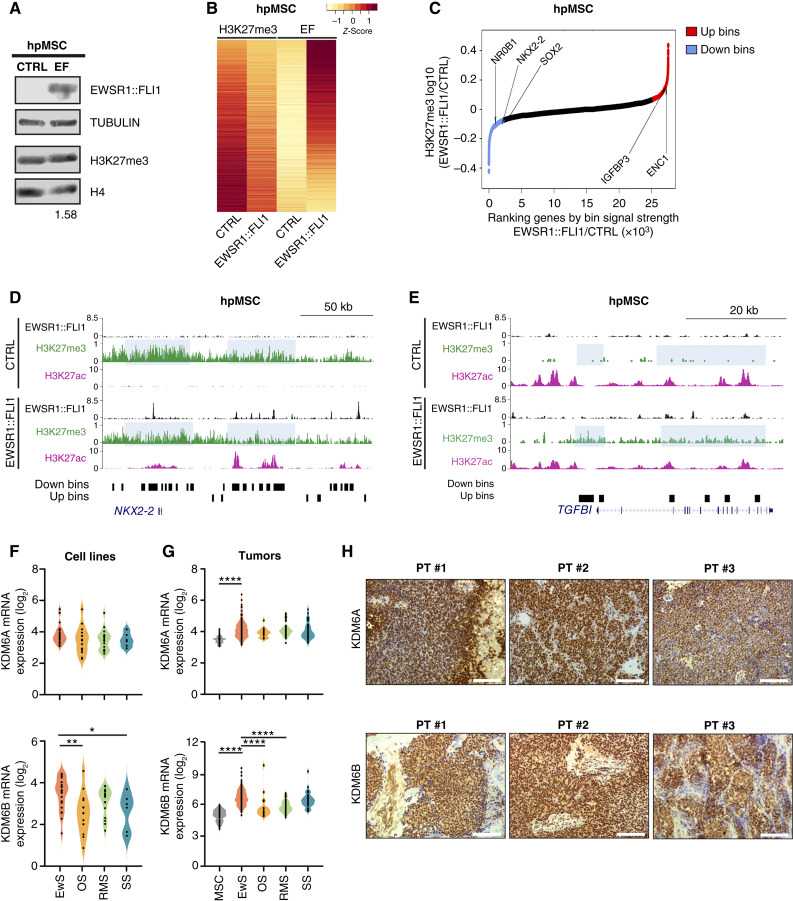
H3K27me3 genome-wide redistribution upon EWSR1::FLI1 overexpression in hpMSCs. **A,** Western blot showing levels of EWSR1::FLI1 and H3K27me3 in whole cell and histone extracts, respectively, in control (CTRL) and upon EWSR1::FLI1 overexpression in hpMSC (EF). Tubulin and histone H4 were used as loading controls. H3K27me3 was quantified relative to H4 and control. **B,** Heatmap depicting H3K27me3 and EWSR1::FLI1 (EF) ChIP-seq in 4,080 H3K27me3-EWSR1::FLI1 coinciding bins for control and EWSR1::FLI1 hpMSC. **C,** Ranking of genes associated with H3K27me3 signal strength in bins (red, genes gaining; blue, genes losing H3K27me3) upon EWSR1::FLI1 overexpression in hpMSC. **D,** UCSC Genome Browser signal tracks for H3K27me3, H3K27ac, and EWSR1::FLI1 in control and EWSR1::FLI1 hpMSC at the *NKX2-2* gene. Up or down bins correspond to regions that gain or lose H3K27me3 upon EWSR1::FLI1 introduction, respectively, and are represented as black rectangles below tracks. Clusters of bins are highlighted in light blue. **E,** As in **D** at the *TGFBI* gene. **F,** Violin plots representing mRNA levels of KDM6A (top) and KDM6B (bottom) in a panel of Ewing sarcoma (EwS), osteosarcoma (OS), rhabdomyosarcoma (RMS), and synovial sarcoma (SS) cell lines extracted from Barretina and colleagues (50). **G,** As in **F**, in primary sarcoma tumors from the NCBI GEO public repository. MSCs derived from healthy bone marrow were used as control tissue. **H,** IHC staining of KDM6A (top) and KDM6B (bottom) on sections of representative primary Ewing sarcoma tumors (PT) from the cohort of 43 tumors from our institution, counterstained with hematoxylin–eosin. Tumor samples are numbered with “#.” White scale bar, 100 µm. Statistical significance was determined by one-way ANOVA test with Holm–Šídák multiple comparison test (**F** and **G**) and relative to Ewing sarcoma. *, *P* < 0.05; **, *P* < 0.01; ****, *P* < 0.0001.

Our group and others have demonstrated that overexpression of EWSR1::FLI1 triggers a loss of H3K27me3 at the promoters of genes that become transcriptionally activated in human umbilical vein endothelial cells and neural crest stem cells (20, 48). In an attempt to determine the extent of such a decrease in H3K27me3 genome-wide, we analyzed published ChIP-seq data on H3K27me3 in hpMSCs overexpressing the fusion oncogene (39). We first examined the distribution of H3K27me3 in genes decorated by this mark in the hpMSC control condition (5 kb around TSS) and revealed a maximal decrease at 2 kb upstream of the TSS upon EWSR1::FLI1 overexpression, concomitant with an increase in H3K27ac specific for the TSS (Supplementary Fig. S1A). Nevertheless, Western blot analysis reported similar H3K27me3 levels in both conditions (Fig. 1A), suggesting an overall genome-wide redistribution following the introduction of EWSR1::FLI1. To confirm this hypothesis, we segmented the genome into 3,069,655 bins of 1 kb and determined that the averaged H3K27me3 signal strength along the bins was highly correlated between control and EWSR1::FLI1 samples, confirming the redistribution of this mark (Supplementary Fig. S1B). Specifically, we identified 103,766 bins with a significant reduction of H3K27me3 (Down bins) and 110,845 bins with a significant gain of H3K27me3 (Up bins) upon EWSR1::FLI1 overexpression (Supplementary Fig. S1C). Of note, as previously proposed (20), most genomic regions presenting H3K27me3 down bins upon the expression of EWSR1::FLI1 in hpMSC are notably enriched over regulatory sequences of bivalent genes (Supplementary Fig. S1D). Integration of H3K27me3 data with ChIP-seq data for EWSR1::FLI1 reveals 67,224 bins in which the oncogene was enriched. Importantly, we observed a progressive loss of H3K27me3 along with a gain of the EWSR1::FLI1 signal in a fraction of 4,080 overlapping bins upon EWSR1::FLI1 overexpression (Fig. 1B). Indeed, the H3K27me3 down bins overlapping with EWSR1::FLI1 and gaining H3K27ac are much higher (32%) than those H3K27me3 up bins overlapping with EWSR1::FLI1 and losing H3K27ac (8%). To test whether H3K27me3 redistribution occurs at the gene level, we searched for those genes in which the H3K27me3 signal upon EWSR1::FLI1 overexpression is significantly changed regardless of the EWSR1::FLI1 signal (FC2, min >0.01; 2,879 genes down and 2,621 genes up; Fig. 1C). Functional analysis of genes losing H3K27me3 correlated with neural processes and metabolism (Supplementary Fig. S1E; Supplementary Table S2). For instance, *NKX2-2*, a well-known EWSR1::FLI1 target, exhibited a gain of H3K27ac with a concomitant moderate H3K27me3 decay along its promoter and regulating enhancers (Fig. 1D). Association with the TGF-beta receptor signaling pathway was obtained for genes gaining H3K27me3 (Supplementary Fig. S1E; Supplementary Table S2), in agreement with the well-known repressed state of genes from this pathway in Ewing sarcoma (49). For instance, *TGFBI* was one of the genes in which a gain of H3K27me3, along with a decrease of H3K27ac after EWSR1::FLI1 introduction, was observed along the gene body (Fig. 1E). Altogether, these results demonstrate that EWSR1::FLI1 targets regions previously decorated with H3K27me3, promoting its redistribution.

Given these observations, we hypothesized that KDM6A and KDM6B, which are the enzymes responsible for demethylating H3K27me3 (24, 25), might be implicated in the loss of H3K27me3 at the promoters and enhancers of EWSR1::FLI1 targets. We therefore evaluated the expression of KDM6A and KDM6B demethylases in a panel of cancer cell lines (50). Although KDM6A expression was similar across tumor types, KDM6B was expressed at higher levels in Ewing sarcoma cell lines than in other sarcomas (Fig. 1F). Among the Ewing sarcoma cell lines, the expression of KDM6A was similar in cell lines containing EWSR1::ERG compared with those containing EWSR1::FLI1, whereas KDM6B showed higher levels in those with EWSR1::FLI1 (Supplementary Fig. S1F). Similar trends were reported in 184 Ewing sarcoma tumors at diagnosis (GSE17679, GSE34620, and GSE37371 accessions), in which we confirmed that the expression of both KDM6A and KDM6B is consistently higher than in MSC (Fig. 1G). KDM6B expression in Ewing sarcoma tumors was particularly high compared with other sarcomas. Analysis of KDM6A and KDM6B by IHC in 43 Ewing sarcoma primary tumor specimens from newly diagnosed patients at our institution revealed that both demethylases were highly expressed by semiquantitative *H*-score analysis (Fig. 1H; Supplementary Fig. S1G). No correlation between KDM6A and KDM6B expression in Ewing sarcoma tumors was found (Supplementary Fig. S1H). Taken together, gene expression data and IHC studies indicate that KDM6A and KDM6B are highly expressed in Ewing sarcoma cell lines and primary tumor samples.

### KDM6A and KDM6B colocalize genome-wide with EWSR1::FLI1 at primed and active enhancers

**Figure 2.**
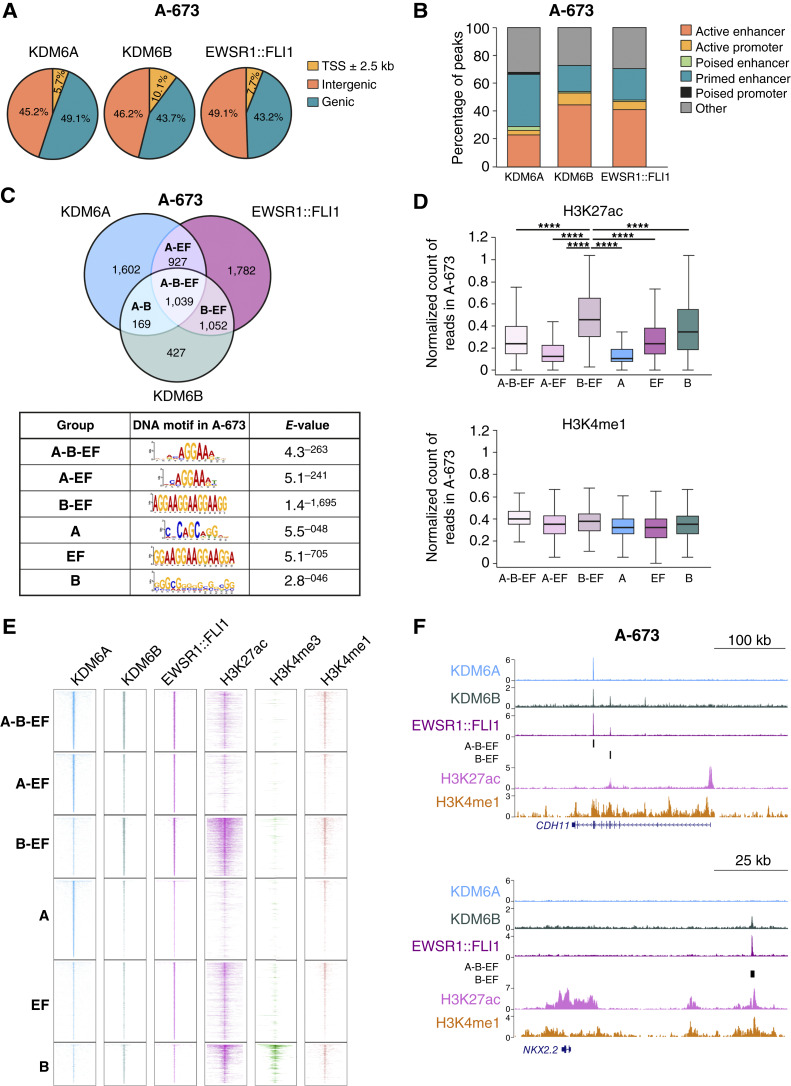
KDM6A and KDM6B colocalize genome-wide with EWSR1::FLI1 at primed and active enhancers. **A,** Pie chart showing the genomic distribution of KDM6A, KDM6B, and EWSR1::FLI1 peaks relative to functional categories, including promoters, genic regions, and intergenic regions in A-673 cells. **B,** Bar plot depicting the percentage of total regulatory elements in the genome overlapping with KDM6A, KDM6B, and EWSR1::FLI1 peaks. **C,** Top, Venn diagram depicting the overlap of KDM6A, KDM6B, and EWSR1::FLI1 peaks in A-673 cells. Bottom, table shows the top MEME DNA motifs and the corresponding *E* value for every group of peaks. **D,** Box plots depicting the average ChIP-seq signal of H3K27ac (top) and H3K4me1 (bottom) on each subset of peaks. **E,** Heatmap of KDM6A, KDM6B, EWSR1::FLI1, H3K27ac, H3K4me3, and H3K4me1 ChIP-seq signals for each group of peaks. **F,** UCSC Genome Browser signal tracks for KDM6A, KDM6B, EWSR1::FLI1, H3K27ac, and H3K4me1 in A-673 cells at the *CDH11* enhancer (top) and the *NKX2-2* enhancer (bottom). EWSR1::FLI1-KDM6B peaks, with or without KDM6A (A-B-EF or B-EF, respectively), are represented as black bars below the tracks. Statistical significance was determined by the Wilcoxon signed-rank test relative to KDM6B-EF peaks (**D**). Error bars in **D**, SD. ****, *P* < 0.0001.

To shed light on whether KDM6A and KDM6B demethylases are involved in H3K27me3 to H3K27ac switches in enhancers regulated by EWSR1::FLI1, we carried out ChIP-seq for KDM6A, KDM6B, and EWSR1::FLI1 in the Ewing sarcoma cell line A-673. We identified 3,737 peaks for KDM6A, 2,687 for KDM6B, and 4,800 for EWSR1::FLI1 (*P* value < 0.05 in all cases; FDR <10^−3^ for KDM6A and EWSR1::FLI1, FDR <10^−5^ for KDM6B; Supplementary Fig. S2A; Supplementary Table S3). At the genomic level, we observed a clear preference for intergenic and genic regions in all cases (Fig. 2A), which indicates a role at enhancers as previously described (27, 29). KDM6B peaks were slightly enriched at promoter regions (10.1%), compared with KDM6A and EWSR1::FLI1 peaks (5.7% and 7.7%, respectively). We next categorized KDM6A, KDM6B, and EWSR1::FLI1 peaks into active, primed, or poised enhancers and into active or poised promoters based on Blanco and colleagues (51). Primed enhancers are regulatory regions that correlate with the H3K4me1 mark in the absence of high levels of H3K27ac (52). Remarkably, KDM6A is more abundant at primed enhancers, whereas KDM6B mimics EWSR1::FLI1 distribution and mainly associates with active enhancers (Fig. 2B). In agreement with this, motif analysis found the characteristic single instance of the GGAA consensus on KDM6A sites, whereas KDM6B and EWSR1::FLI1 peaks were enriched in both multiple and single GGAA motifs (Supplementary Fig. S2B). Strikingly, when intersecting KDM6A and KDM6B peaks with the set of 697 EWSR1::FLI1 superenhancers defined by Tomazou and colleagues (17), we found that 23% of superenhancers overlap with KDM6A and 35% with KDM6B. To elucidate the extent to which KDM6A and KDM6B colocalize with EWSR1::FLI1, we intersected peaks from the three entities and found a strong overlap of EWSR1::FLI1 with both demethylases (A-B-EF group) and with each demethylase separately (A-EF and B-EF; Fig. 2C). Gene association to peaks retrieved 1,511 genes for KDM6A and 1,207 genes for KDM6B, with 921 genes being direct EWSR1::FLI1-KDM6A targets and 893 for EWSR1::FLI1-KDM6B. These targets were enriched in GO categories related to axon guidance, axonogenesis, or neuron projection for both demethylases (Supplementary Fig. S2C; Supplementary Table S4), highlighting their important role in promoting neuronal differentiation in Ewing sarcoma and consistent with previous findings in neuronal progenitors (26). Primed enhancers define a state prior to activation that does not yield RNA and correlates with cell type specificity (52, 53). Thus, the enrichment of KDM6A in primed enhancers supports the involvement of this enzyme in neural cell specification. The intersection obtained in A-673 was validated in a second Ewing sarcoma cell line, TC-71, in which we found 1,163 common peaks between EWSR1::FLI1 and KDM6A/KDM6B (*P* value < 0.05; FDR <10^−5^ in all cases; Supplementary Fig. S2D). Of note, ChIP-seq for EZH2 retrieves no overlap with EWSR1::FLI1 (Supplementary Fig. S2E), in agreement with previous studies (16, 20). Motif analysis in the A-673 cell line revealed a strong enrichment in GGAA multimeric repeats when EWSR1::FLI1 stands alone or together with KDM6B (B-EF group), whereas the presence of KDM6A is linked to the single GGAA motif (A-B-EF and A-EF groups), suggesting that KDM6A partners with a unique set of EWSR1::FLI1 peaks (Fig. 2C). In agreement with our motif characterization, KDM6B, together with EWSR1::FLI1 (B-EF group), strongly overlaps with high levels of H3K27ac and H3K4me1 (i.e., active enhancers), whereas KDM6A (A-B-EF and A-EF groups) is associated with lower levels of H3K27ac and enrichment in H3K4me1 (i.e., primed enhancers; Fig. 2D and E). No significant enrichment of H3K27me3 was found in any collection of peaks (Supplementary Fig. S2F). Remarkably, KDM6B alone was found in a subset of transcriptionally active promoters (H3K4me3 and H3K27ac; Fig. 2E; Supplementary Fig. S2F), suggesting differential roles for each demethylase. Indeed, analysis of the DepMap database (https://depmap.org/portal) retrieves negative Chronos scores for KDM6B in A-673 and TC-71 Ewing sarcoma cell lines, whereas KDM6A does not constitute a dependency in these cells (−0.05 and −0.15; 0.04 and 0.013, respectively), supporting the correlation of KDM6B with GGAA repeats. Overall, our data support three classes of enhancers according to their composition: (i) those in which KDM6A and KDM6B colocalize with the oncogene (A-B-EF) at single GGAA motifs (e.g., *CDH11* gene, Fig. 2F; Supplementary Fig. S2G), (ii) EWSR1::FLI1-KDM6A enhancers (without KDM6B, A-EF) containing single GGAA motifs (e.g., *SMYD3*, Supplementary Fig. S2H), and (iii) those containing the overlap of KDM6B and EWSR1::FLI1 (without KDM6A, B-EF) and enrichment at multimeric GGAA repeats (e.g., *NKX2-2* enhancer, Fig. 2F; Supplementary Fig. S2G). Notably, the colocalization of KDM6A and KDM6B at EWSR1::ERG-bound enhancer regions was also observed in the RM-82 cell line (Supplementary Fig. S2I). The differential location and partnership identified for each of the KDM6 demethylases in Ewing sarcoma suggest that KDM6B specifies EWSR1::FLI1 for the most active regions and KDM6A signals for a specific set of enhancers that commit to neural lineage.

### Knockdown of KDM6A and KDM6B downregulates EWSR1::FLI1-activated targets

**Figure 3.**
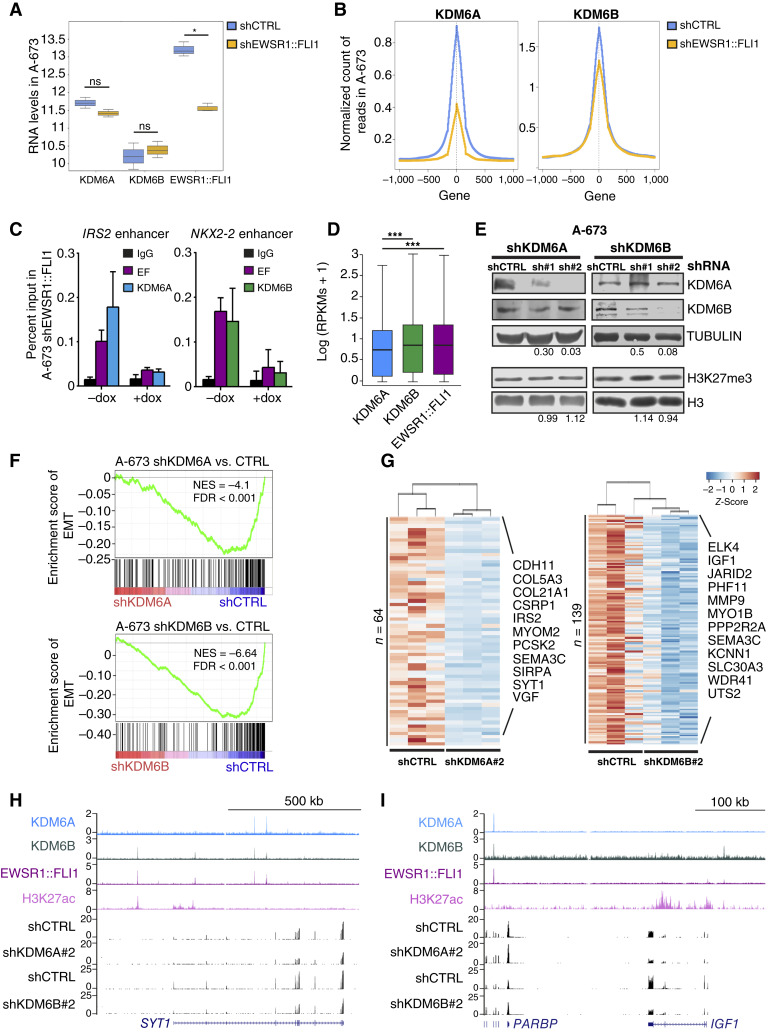
Knockdown of KDM6A and KDM6B downregulates EWSR1::FLI1-activated targets. **A,** Box plot representing the RNA levels of *KDM6A*, *KDM6B*, and *EWSR1::FLI1* in control and EWSR1::FLI1 knockdown in A-673 Ewing sarcoma cells from Orth and colleagues (45). **B,** Metagene plot showing KDM6A (left) and KDM6B (right) ChIP-seq signals in 3,634 and 1,000 peaks, respectively, at TSS (0) and within a 1,000 kb window in control (shCTRL) and upon doxycycline induction of EWSR1::FLI1 knockdown in A-673 (shEWSR1::FLI1). **C,** ChIP-qPCR of EWSR1::FLI1 (EF), KDM6A (left) and KDM6B (right) enrichment at the enhancer region of *IRS2* and *NKX2-2* genes, respectively, upon doxycycline-induced knockdown (+dox) of the fusion in A-673 cells. **D,** Box plot representing the RNA levels in A-673 cells of KDM6A, KDM6B, and EWSR1::FLI1 target genes from Riggi and colleagues (16). RPKM, reads per kilobase of transcript per million mapped reads. **E,** Western blot showing levels of KDM6A and KDM6B in whole cell extracts (top) and H3K27me3 in histone extracts (bottom) upon KDM6A (left) or KDM6B (right) knockdown with two shRNA sequences (sh#1 and sh#2) at 72 hours of doxycycline induction in A-673 cells. Tubulin and H3 were used as loading controls. Levels were quantified relative to tubulin/H3 and control. **F,** Gene set enrichment analysis curves and normalized enrichment scores (NES) for the Hallmark collection of epithelial-to-mesenchymal transition (EMT) upon shKDM6A#2 (top) and shKDM6B#2 (bottom) targets in A-673 cells. FDR is also shown. **G,** Heatmap and dendrogram showing expression levels of genes in the vicinity (100 kb) of EWSR1::FLI1-KDM6A (left) and EWSR1::FLI1-KDM6B (right) ChIP-seq peaks significantly downregulated upon knockdown of each demethylase in A-673 cells. *n* indicates the number of deregulated direct targets upon knockdown. **H,** UCSC Genome Browser signal tracks for KDM6A, KDM6B, EWSR1::FLI1, and H3K27ac at the *SYT1* gene. RNA-seq tracks for shKDM6A (shRNA#2) and shKDM6B (shRNA#2) and the corresponding shCTRL in A-673 are shown. **I,** As in **H**, at the *IGF1* gene. Statistical differences between groups were assessed by the Student *t* test (**A**), Mann–Whitney *t* test (**C**), and Wilcoxon signed-rank test (**D**). Error bars in **A**, **C**, and **D** indicate SD. *, *P* < 0.05; ***, *P* < 0.001; ns, not significant.

To understand whether EWSR1::FLI1 affects the distribution of KDM6A and KDM6B on chromatin, we performed KDM6A and KDM6B ChIP-seq analysis in an A-673 cell line with inducible knockdown of EWSR1::FLI1 (42). As shown in Fig. 3A, the knockdown of the oncogene does not alter KDM6 expression (45), whereas the recruitment of both KDM6A and KDM6B is decreased genome-wide (Fig. 3B). Furthermore, we confirmed the loss of both demethylases at EWSR1::FLI1 targets upon knockdown of the oncogene (Fig. 3C). These data support that EWSR1::FLI1 is responsible for the recruitment of the demethylases. Our previous results pointed to two different classes of EWSR1::FLI1-induced neoenhancers when partnered with KDM6A—at primed enhancers—or with KDM6B—at active enhancers. Indeed, the expression of genes in the proximity of enhancers containing KDM6A is lower than that of those with KDM6B (Fig. 3D). To gain insight into the role of both demethylases on the EWSR1::FLI1 transactivation activity, we knocked down KDM6A and KDM6B in two Ewing sarcoma cell lines, A-673 and TC-71, using a doxycycline-inducible system. We confirmed the knockdown of the demethylases at the protein level with two different shRNA sequences (KDM6A sh#1 and sh#2; KDM6B sh#1 and sh#2) in both A-673 and TC-71 cells, whereas the evaluation of H3K27me3 global levels in the KDM6B knockdown showed no noticeable changes (Fig. 3E; Supplementary Fig. S3A). To study genome-wide expression changes, we carried out a global transcriptome analysis by RNA-seq upon the knockdown of KDM6A (sh#1 and sh#2) and KDM6B (sh#1 and sh#2). Loss of each demethylase resulted in gene downregulation in A-673 [71.4% and 68% for KDM6A; 72.5% and 67.8% for KDM6B; adjusted *P* < 0.01 and log-fold change (FC) cutoff = 0.5 and fragments per kilobase of transcript per million mapped reads (FPKM) >10; Supplementary Fig. S3B; Supplementary Table S5], and this tendency was confirmed in the TC-71 cell line (61.7% and 59.4% for KDM6A; 38.1% and 56.1% for KDM6B; adjusted *P* < 0.1 and log FC cutoff = 0.32 and FPKM >5; Supplementary Table S5). Gene set enrichment analysis in the A-673 cell line found that both KDM6A and KDM6B are necessary for the activation of epithelial-to-mesenchymal transition genes (Fig. 3F; Supplementary Table S5). However, there is little overlap between both sets of deregulated genes upon demethylase knockdown (86 upregulated and 34 downregulated genes in common between KDM6A and KDM6B), confirming different functionalities for each demethylase. All these results support the selective active role of KDM6A and KDM6B in the transcriptional activation network necessary for maintaining the EWSR1::FLI1 oncogenic signature.

To further understand the relevance of KDM6A and KDM6B in the transcriptional activation program of EWSR1::FLI1, we intersected genes associated with the genomic regions in which these demethylases and EWSR1::FLI1 colocalize with deregulated targets in A-673. To associate genes with peaks, we captured every gene within 100 kb of both combinations of ChIP-seq peaks and obtained 3,134 genes and 3,326 genes in the vicinity of EWSR1::FLI1-KDM6A and EWSR1::FLI1-KDM6B, respectively. We then intersected these direct targets with the set of KDM6A and KDM6B significantly downregulated or upregulated genes. We obtained 64 directly activated targets for KDM6A and 139 for KDM6B with the EWSR1::FLI1 peak (Fig. 3G; Supplementary Table S5), whereas 20 directly repressed targets for KDM6A and 101 for KDM6B were identified (Supplementary Fig. S3C). GO analysis of activated targets reported neural categories such as axonogenesis, previously observed in Supplementary Fig. S2C, and cell migration (Supplementary Fig. S3D). Among the list of EWSR1::FLI1-KDM6A–activated targets, we found *CDH11*, *IRS2*, and *SYT1* (Fig. 3G and H; Supplementary Fig. S3E), whereas for EWSR1::FLI1-KDM6B, we found targets such as *IGF1*, *MMP9*, and *JARID2*, previously described in Ewing sarcoma (Fig. 3G and I; Supplementary Fig. S3F). A decrease in specific KDM6A and KDM6B targets was also observed in the RM-82 cell line (Supplementary Fig. S3G). Interestingly, most of the direct targets of KDM6A contain peaks for KDM6B although only a few targets were commonly downregulated. These results support the differential role of KDM6A and KDM6B in transcriptional activation in Ewing sarcoma.

### KDM6A recruits BRG1 to EWSR1::FLI1-activated enhancers in a demethylase-independent manner

**Figure 4.**
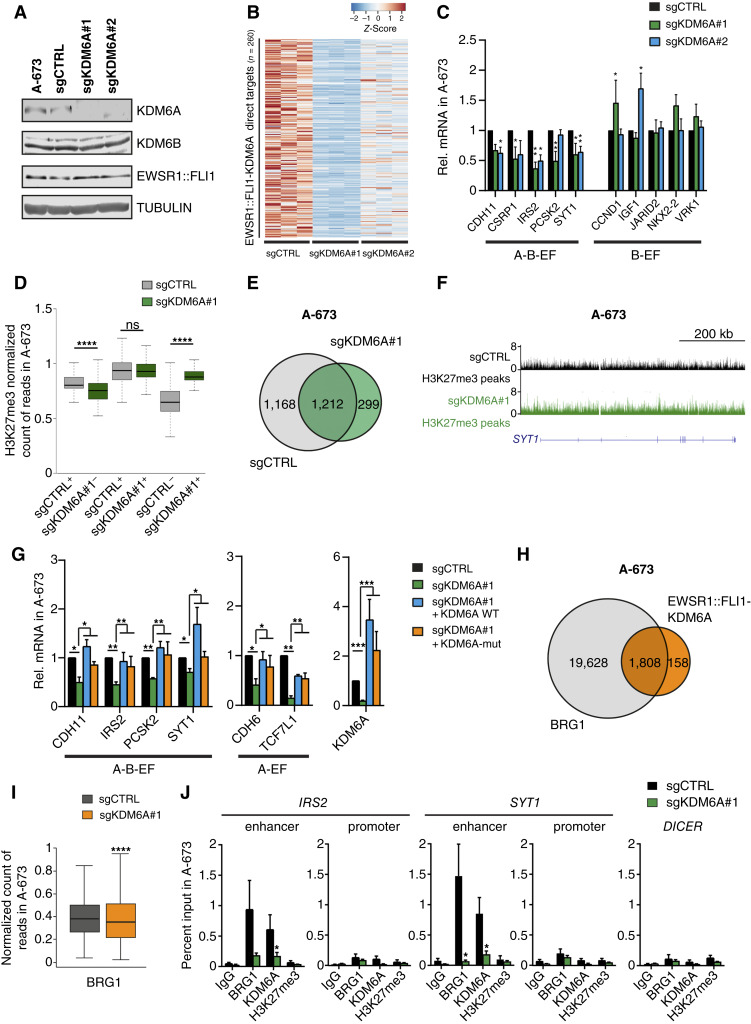
KDM6A recruits BRG1 to EWSR1::FLI1-activated enhancers in a demethylase-independent manner. **A,** Western blot of KDM6A, KDM6B, and EWSR1::FLI1 in whole cell extracts upon KDM6A KO with two sgRNA sequences (#1 and #2) in A-673 cells. Tubulin was used as a loading control. **B,** Heatmap showing expression levels of significantly downregulated genes in the vicinity (100 kb) of EWSR1::FLI1-KDM6A ChIP-seq peaks upon sgKDM6A#1 and sgKDM6A#2 in A-673 cells. **C,** RT-qPCR of EWSR1::FLI1-KDM6B targets with or without KDM6A ChIP-seq peaks in A-673 sgKDM6A#1 and sgKDM6A#2. **D,** Box plot depicting the average ChIP-seq signal of H3K27me3 at 4,369 peaks exclusive to sgCTRL or 1,616 peaks for sgKDM6A#1 (sgCTRL^+^/sgKDM6A^−^ and sgCTRL^−^/sgKDM6A^+^, respectively) and 1,610 peaks in common (sgCTRL^+^/sgKDM6A^+^). **E,** Venn diagram depicting genes associated with H3K27me3 peaks in sgCTRL and sgKDM6A#1. **F,** UCSC Genome Browser signal tracks for H3K27me3 at the *SYT1* intronic enhancer in sgCTRL and sgKDM6A#1. **G,** RT-qPCR of EWSR1::FLI1-KDM6A targets with (left) or without KDM6B (middle) in A-673 sgCTRL+ empty vector (sgCTRL), sgKDM6A#1 + empty vector (sgKDM6A#1), and upon overexpression of KDM6A WT (sgKDM6A#1 + KDM6A WT) and the dead mutant (H1146A/E1148A; sgKDM6A#1 + KDM6A-mut) forms in sgKDM6A#1. **H,** Venn diagram depicting the overlap between EWSR1::FLI1-KDM6A and BRG1 peaks in A-673 cells. **I,** Box plot of BRG1 ChIP-seq signal on the common set of 1,808 EWSR1::FLI1-KDM6A and BRG1 peaks upon sgKDM6A#1 in A-673. **J,** ChIP-qPCR of BRG1, KDM6A, and H3K27me3 enrichment at the enhancer and promoter regions of *IRS2* and *SYT1* KDM6A-activated targets upon sgKDM6A#1. *DICER* was used as a negative control region. Kruskal–Wallis test with Dunn correction relative to sgCTRL (**C** and **I**), Wilcoxon signed-rank test (**D**), two-tailed Mann–Whitney test relative to sgKDM6A#1 (**G**), and ordinary two-way ANOVA with Holm–Šídák test relative to control (**J**) were applied. *GAPDH* was used as a housekeeping gene (**C** and **G**). Error bars indicate SEM (**C**, **G**, and **J**) of three independent biological experiments and SD (**D** and **I**). *, *P* < 0.05; **, *P* < 0.01; ***, *P* < 0.001;****, *P* < 0.0001; ns, not significant.

To avoid off-target effects and induce permanent gene modification, we generated KOs of KDM6 using CRISPR methodology. We confirmed that our KO completely abolished KDM6A expression in A-673 cells without causing changes in EWSR1::FLI1 and KDM6B levels (Fig. 4A). Despite the loss of demethylase enzymatic activity, global levels of H3K27me3 as well as H3K4me1 and H3K27ac were not altered upon KO in A-673 (Supplementary Fig. S4A). Further characterization of KDM6A KO by RNA-seq showed that the percentage of downregulated genes (activated targets, 69.1% and 67.1% for sgRNA#1 and sgRNA#2, respectively) was higher than for upregulated genes (repressed targets, 30.9% and 32.9% for sgRNA#1 and sgRNA#2, respectively), supporting the transcriptional activation role of KDM6A. Indeed, activated genes were enriched in axonogenesis categories (Supplementary Fig. S4B) as already observed with shRNA (Supplementary Fig. S3D). Intersection of EWSR1::FLI1-KDM6A ChIP-seq direct targets (distance of 100 kb) with significantly decreased targets for KDM6A KO confirmed 260 direct activated targets (adjusted *P* < 0.05 and log FC cutoff = 1, Fig. 4B; Supplementary Table S6), enriched in the same categories as the whole set of KDM6A targets (Supplementary Fig. S4B). We further validated by RT-qPCR the downregulation of genes containing KDM6A and EWSR1::FLI1 with KDM6B (A-B-EF group), whereas the expression of targets containing KDM6B and EWSR1::FLI1 (B-EF group) remained unchanged (Fig. 4C). The decrease in gene expression of EWSR1::FLI1-KDM6A targets identified by ChIP-seq (Supplementary Fig. S2D) was confirmed in a second Ewing sarcoma cell line, TC-71, upon KDM6A KO (Supplementary Fig. S4C and S4D).

To elucidate genome-wide, the contribution of KDM6A demethylase activity in the decreased expression of defined targets, we performed H3K27me3 ChIP-seq normalized with spike-in in A-673 control and KDM6A KO (sgCTRL and sgKDM6A A-673 cells, respectively). The resulting H3K27me3 peaks from each condition were intersected and divided into three classes: (i) commonly found in control and KDM6A KO (sgCTRL+/sgKDM6A+), (ii) only found in the control condition (sgCTRL+/sgKDM6A−), and (iii) only found in KDM6A KO (sgCTRL−/sgKDM6A+). Quantification of signal strength for each class of peaks showed no changes in the common group, harboring the most intense peak signals from repressed regions, whereas a new group of 1,616 peaks emerged upon deletion of the demethylase (Fig. 4D). Those regions gaining H3K27me3 upon KDM6A KO are associated with 299 genes (Fig. 4E). However, the average profile of H3K27me3 around the TSS of KDM6A-activated genes (downregulated upon KDM6A KO) revealed no correlation between changes in their gene expression and H3K27me3 levels (Supplementary Fig. S4E), exemplified by *SYT1* in Fig. 4F. To more accurately pinpoint the regions of the genome presenting higher H3K27me3 changes, we segmented the genome into bins of 1 kb and determined the average signal strength of the ChIP-seq for both conditions. This analysis retrieved 579,992 bins gaining (Up) H3K27me3 and 142,556 bins losing H3K27me3 (Down; Supplementary Fig. S4F and S4G), supporting a general redistribution of the mark.

KDM6A demethylase-independent functions have been proposed in Kabuki syndrome, in the context of cell-induced differentiation with retinoic acid, mesoderm differentiation of embryonic stem cells, and in lung cancer (28, 35, 43, 54). To analyze the contribution of KDM6A demethylase activity at EWSR1::FLI1-KDM6A targets, we reintroduced KDM6A WT or a dead mutant enzyme (mutations in H1146A and E1148A) in KDM6A KO cells (43). As shown in Fig. 4G, both WT and dead mutant enzymes recovered the expression of EWSR1::FLI1-KDM6A target genes (*CDH11*, *IRS2*, *PCSK2*, and *SYT1*), confirming that the transcriptional activating function of KDM6A is independent of its demethylase activity. This recovery of expression was also observed in genes in which only KDM6A is present with the oncogene, such as *CDH6* and *TCF7L1* (Fig. 4G).

KDM6A has been described to physically associate with the BAF complex member BRG1/SMARCA4 (55, 56), which constitutes the central catalytic subunit that uses the energy derived from ATP hydrolysis to remodel nucleosomes and regulate transcription (57). To elucidate whether KDM6A was mediating transcriptional activation at enhancers through the recruitment of BRG1, we next evaluated BRG1 enrichment genome-wide by ChIP-seq. The intersection of EWSR1::FLI1-KDM6A with BRG1 at the peak level retrieved 1,808 common regions (Fig. 4H), in which BRG1 ChIP-seq signal significantly decreased upon KDM6A KO (Fig. 4I). Validation of genome-wide results by ChIP-qPCR upon KDM6A KO showed a decrease in BRG1 at enhancers of *IRS2* and *SYT1* EWSR1::FLI1-KDM6A–activated target genes, whereas H3K27me3 levels were unmodified both at enhancer and promoter regions in A-673 and TC-71 cell lines (Fig. 4J; Supplementary Fig. S4H). Overexpression of both WT and dead mutant in KDM6A KO cells rescued the levels of BRG1 at the *SYT1* enhancer (Supplementary Fig. S4I). Altogether, our results suggest that KDM6A contributes to the recruitment of BRG1 at EWSR1::FLI1–activated enhancers, facilitating transcriptional activation through a demethylase-independent mechanism.

### KDM6A KO decreases Ewing sarcoma tumor growth

**Figure 5.**
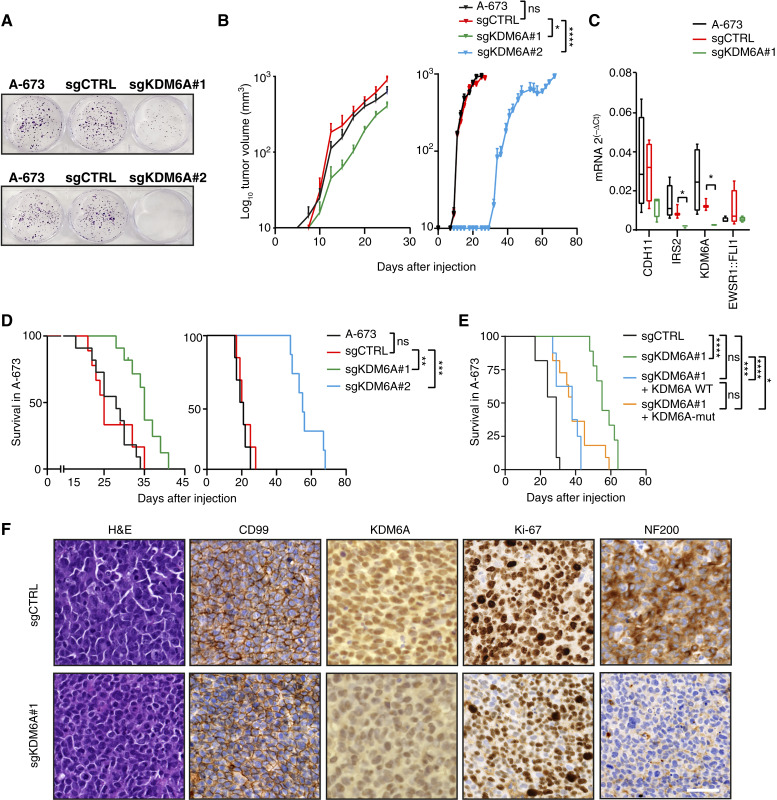
KDM6A KO decreases Ewing sarcoma tumor growth. **A,** Colony formation assay of KDM6A KO cells (sgRNA#1 and sgRNA#2) compared with parental cells and sgCTRL in A-673. **B,** Tumor growth curves of the average volume of xenografts derived from subcutaneous injection of KDM6A KO cells compared with parental and sgCTRL A-673 cells in nude athymic mice (left, *n* = 11 for parental, *n* = 11 for sgKDM6A#1, and *n* = 9 for sgCTRL; right, *n* = 9 for parental, *n* = 7 for sgKDM6A#2, and *n* = 12 for sgCTRL; *n*, number of tumors). **C,** RT-qPCR determination of KDM6A targets *CDH11* and *IRS2* in RNA extracts from four tumors excised from parental, sgCTRL, and sgKDM6A (sgRNA#1) xenografts of A-673 cells. *EWSR1::FLI1* and *KDM6A* expression are also shown. *GAPDH* was used as a housekeeping gene. **D,** Kaplan–Meier curve representing the percentage of xenograft tumors that reach a tumor volume of 1,000 mm^3^ within days after injection in parental, sgCTRL, and KDM6A KO (sgRNA#1 and sgRNA#2) in A-673 cells. **E,** As in **D**, representing the percentage of xenograft tumors that reach a tumor volume of 1,500 mm^3^ in A-673 sgCTRL+ empty vector (sgCTRL; *n* = 11), sgKDM6A#1 + empty vector (sgKDM6A#1; *n* = 9), and upon overexpression of KDM6A WT (sgKDM6A#1 + KDM6A WT; *n* = 8) and the dead mutant (H1146A/E1148A; sgKDM6A#1 + KDM6A-mut; *n* = 11) in sgKDM6A#1. **F,** IHC staining of CD99, KDM6A, Ki-67, and NF200 on sections of tumors excised from sgCTRL and sgKDM6A (sgRNA#1) xenografts of A-673 cells. White scale bar, 50 µm. H&E, hematoxylin and eosin. Statistical significance was determined by repeated measures two-way ANOVA (**B**) and the Kruskal–Wallis test with Dunn multiple comparison correction (**C**) relative to sgCTRL. Survival analysis was performed using the log-rank (Mantel–Cox) test (**D** and **E**) relative to sgCTRL (**D**) and to sgCTRL and sgKDM6A#1 (**E**). Error bars, SEM (**B** and **C**). *, *P* < 0.05; **, *P* < 0.01; ***, *P* < 0.001; ****, *P* < 0.0001; ns, not significant.

Based on the previous results in which we showed KDM6A actively collaborating with EWSR1::FLI1 at enhancers, we aimed to investigate the dependency of Ewing sarcoma tumor formation on KDM6A. First, we observed that KDM6A KO cells exhibited a significant decrease in their clonogenic capacity compared with control and parental cells (Fig. 5A; Supplementary Fig. S5A). Next, we subcutaneously injected sgCTRL, sgKDM6A#1, and sgKDM6A#2 KO A-673 cells into athymic nude mice and monitored tumor growth. Xenografts of parental cells were included as an additional control for tumor growth. Interestingly, we found that KDM6A KO (#1 and #2) showed a delay in tumor growth compared with control and parental-derived tumors (Fig. 5B; Supplementary Fig. S5B). At 17 days after injection, tumors were significantly smaller for sgKDM6A#1 (mean tumor volume of 121 mm^3^ for sgKDM6A#1 and 252.5 and 280 mm^3^ for A-673 and sgCTRL tumors, respectively; Supplementary Fig. S5C). We confirmed the downregulation of KDM6A by RT-qPCR and Western blot (Fig. 5C; Supplementary Fig. S5D). Moreover, median survival increased from 25 days (control) to 35 days for sgKDM6A#1 and from 20.5 days (control) to 55 days for sgKDM6A#2, respectively (Fig. 5D). To understand the contribution of KDM6A demethylase activity in tumor growth, we performed colony formation assays reintroducing KDM6A WT and the dead mutant in KDM6A KO. In both conditions, the number of colonies was recovered at the same level (Supplementary Fig. S5E and S5F). Furthermore, we observed that the dead mutant rescued the tumorigenic phenotype similar to KDM6A WT, decreasing the median survival of KDM6A KO from day 57 to day 39 for both conditions (Fig 5E), whereas tumor growth was partially recovered (Supplementary Fig. S5G). These results support that KDM6A’s contribution to tumor maintenance is independent of demethylase activity. Importantly, we confirmed a decreased expression of enhancer-bound EWSR1::FLI1-KDM6A targets, *CDH11* and *IRS2* (Fig. 5C), in KDM6A KO xenografted tumors, supporting the transcriptional activating role of KDM6A at these oncogenic targets. IHC analysis of tumors confirmed the expression of the Ewing sarcoma marker CD99 and significantly lower expression of KDM6A and the proliferation marker Ki-67 in sgKDM6A-derived tumors (Fig. 5F; Supplementary Fig. S5H and S5I). All these results confirm KDM6A as a critical factor for Ewing sarcoma tumor growth, irrespective of its demethylase activity.

Previously, we found that KDM6A targets are enriched in categories related to neural differentiation (Supplementary Fig. S3D). The heavy chain of neurofilament protein NF200 is highly expressed in Ewing sarcoma cells and tumors (58, 59). Indeed, NF200 is highly expressed in publicly available datasets of Ewing sarcoma tumors (Supplementary Fig. S5J). Furthermore, IHC analysis revealed lower levels of NF200 in KDM6A-KO tumors compared with those from the control group (Fig. 5F). Altogether, our data indicate that KDM6A exerts a critical role in Ewing sarcoma tumor growth and regulates neural pathways *in vivo*.

### Impact of KDM6B KO in EWSR1::FLI1 targets

**Figure 6.**
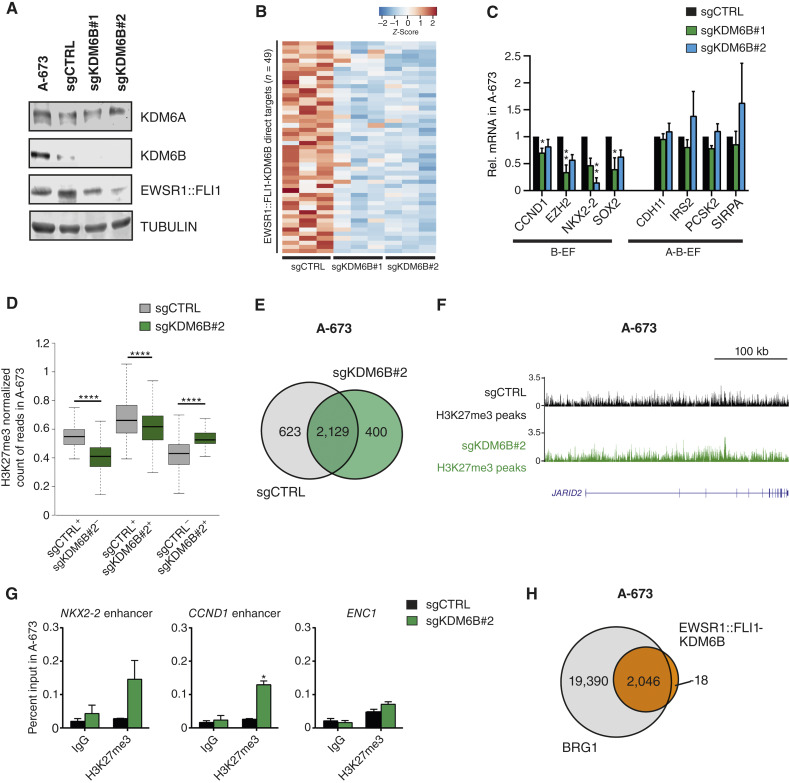
Impact of KDM6B KO on EWSR1::FLI1 targets. **A,** Western blot showing levels of KDM6A, KDM6B, and EWSR1::FLI1 in whole cell extracts upon KDM6B KO with two sgRNA sequences (#1 and #2) in A-673 cells. Tubulin was used as a loading control. **B,** Heatmap showing expression levels of significantly downregulated genes in the vicinity (100 kb) of EWSR1::FLI1-KDM6B ChIP-seq peaks upon sgKDM6B#1 and sgKDM6B#2 in A-673 cells. **C,** RT-qPCR of EWSR1::FLI1-KDM6B targets with or without KDM6A ChIP-seq peaks in A-673 sgKDM6B#1 and sgKDM6B#2. *GAPDH* was used as a housekeeping gene. **D,** Box plot depicting the average ChIP-seq signal of H3K27me3 at 3,248 peaks exclusive to sgCTRL or 2,366 peaks for sgKDM6B#2 (sgCTRL^+^/sgKDM6B^−^ and sgCTRL^−^/sgKDM6B^+^, respectively) and 3,473 peaks in common (sgCTRL^+^/sgKDM6B^+^). **E,** Venn diagram depicting the overlap between genes associated with H3K27me3 peaks in sgCTRL and sgKDM6B#2. **F,** UCSC Genome Browser signal tracks for H3K27me3 at the intronic enhancer of *JARID2* in sgCTRL and sgKDM6B#2. **G,** ChIP-qPCR of H3K27me3 enrichment at the enhancer region of *NKX2-2* and *CCND1* genes upon KDM6B KO with sgRNA#2. *ENC1* was used as a negative control region. **H,** Venn diagram depicting the overlap of EWSR1::FLI1-KDM6B and BRG1 peaks in A-673 cells. Statistical significance was determined by the Kruskal–Wallis test with Dunn multiple comparison correction relative to sgCTRL (**C**), Wilcoxon signed-rank test (**D**), and ordinary two-way ANOVA with Dunnett multiple comparisons test relative to IgG (**G**). Error bars indicate SEM (**C** and **G**) of three independent biological experiments or SD (**D**).  *, *P* < 0.05; **, *P* < 0.01; ****, *P* < 0.0001.

To understand whether the function of KDM6B in Ewing sarcoma cells is associated with its demethylase activity, we investigated the H3K27me3 dynamics in KDM6B-depleted cells. First, we confirmed the KO of KDM6B in A-673 cells (Fig. 6A). Next, we evaluated the levels of H3K27me3 by Western blot in KDM6B KO and found that the depletion of the demethylase did not alter the global levels of H3K27me3 (Supplementary Fig. S6A). Further characterization of KDM6B KO by RNA-seq retrieved higher percentages of downregulated genes (66.8% and 50.6%, activated targets) than upregulated genes (33.3% and 49.4%, repressed targets) for sgRNA#1 and sgRNA#2, respectively, with activated genes enriched in cell migration (Supplementary Fig. S6B). By intersecting EWSR1::FLI1-KDM6B ChIP-seq direct targets (distance of 100 kb) with significantly decreased targets for KDM6B KO, we defined 49 direct activated targets (adjusted *P* < 0.05 and log FC cutoff = 0.5; Fig. 6B; Supplementary Table S6), enriched in axonogenesis and myosin structure (Supplementary Fig. S6B). We confirmed by RT-qPCR that the expression of targets potentially regulated by enhancers containing KDM6B and EWSR1::FLI1 (B-EF group) was dependent on KDM6B, whereas KDM6B KO did not affect the expression of targets containing enhancers with KDM6A binding sites (A-B-EF group; Fig. 6C). Decreased expression of EWSR1::FLI1-KDM6B targets identified by ChIP-seq (Supplementary Fig. S2D) was confirmed in a second cell line, TC-71, upon KDM6B KO (Supplementary Fig. S6C and S6D). To inspect for the demethylase activity of KDM6B genome-wide, we performed H3K27me3 ChIP-seq normalized with spike-in in A-673 control and KDM6B KO (sgCTRL and sgKDM6B, respectively). The obtained peaks were divided into three groups, as previously described for KDM6A. Interestingly, in this case, we observed a decrease in the peaks from the common group (sgCTRL+/sgKDM6B+ group), which are the highest peaks corresponding to repressed regions maintained between both conditions (Fig. 6D). Although unexpected, these results are in concordance with the decrease in EZH2 expression observed upon KDM6B KO (Fig. 6C). As shown in Fig. 6D, H3K27me3 was established in 2,366 peaks from the sgCTRL−/sgKDM6B+ group, corresponding to 400 genes (Fig. 6E), whereas the average profile of H3K27me3 around the TSS of KDM6B-activated genes (downregulated upon KDM6B KO) showed no correlation between changes in their gene expression and H3K27me3 levels (Supplementary Fig. S6E). The intersection of the set of 400 genes gaining H3K27me3 with the 299 genes gaining the repressive mark upon KDM6A loss yielded a minimal overlap of 5%, supporting the differential contribution of each demethylase. Bin analysis of H3K27me3 in both samples (sgCTRL and sgKDM6B#2) resulted in 380,371 bins gaining and 442,200 losing H3K27me3, suggesting a redistribution of the repressive mark (Supplementary Fig. S6F and S6G). Although changes in expression did not correlate with an increase of H3K27me3 at TSS upon KDM6B KO, we observed by ChIP-seq moderate recoveries of the H3K27me3 levels at specific EWSR1::FLI1-KDM6B enhancers, such as *JARID2* (Fig. 6F). Enrichment of the repressive mark at these specific regions was validated by ChIP-qPCR (Fig. 6G). Moreover, when we treated Ewing sarcoma cells with the KDM6A/KDM6B demethylase inhibitor GSK-J4, we found that the expression of EWSR1::FLI1-activated targets decreased strongly in those targets containing only KDM6B, suggesting that GSK-J4 phenocopies expression changes induced by KDM6B deletion (Supplementary Fig. S6H). In accordance with published data describing BAF complex enrichment at GGAA repeats, we show EWSR1::FLI1-KDM6B colocalization with the BRG1 subunit (Fig. 6H), which is enriched in both single and GGAA repeats (Supplementary Fig. S6I). Overall, our results confirm that KDM6B exerts its function as a demethylase at specific EWSR1::FLI1-KDM6B targets, whereas genome-wide changes in H3K27me3 are counterbalanced by the decrease of EZH2 in KDM6B-depleted cells.

### KDM6B KO decreases tumor growth

**Figure 7.**
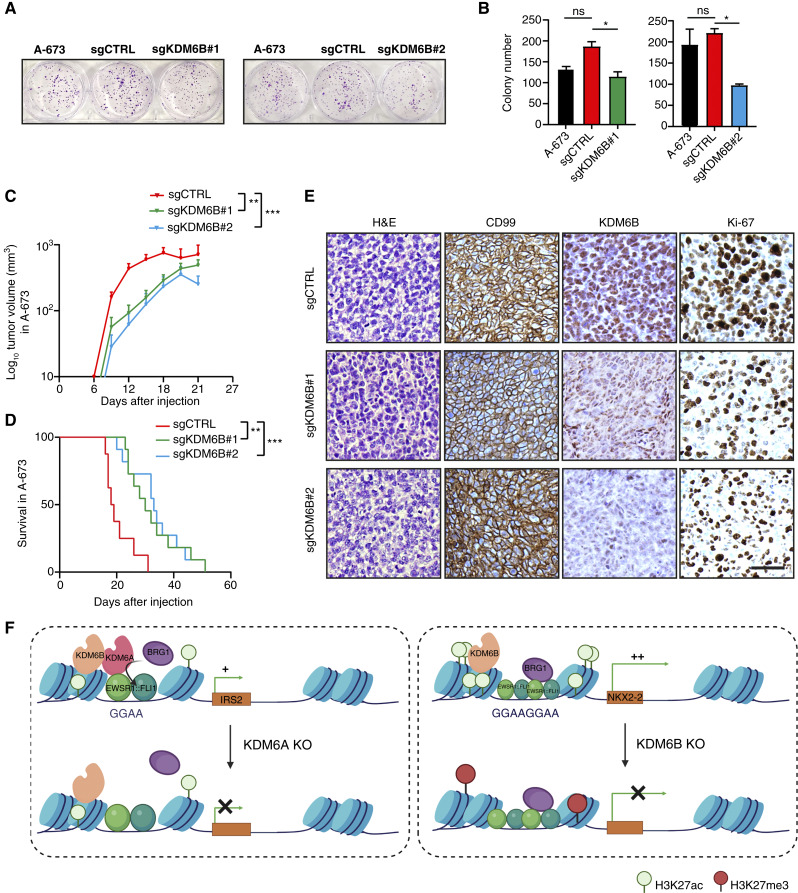
KDM6B KO decreases tumor growth. **A,** Colony formation assay of KDM6B KO cells (sgRNA#1 and sgRNA#2) compared with parental and sgCTRL A-673 cells. **B,** Bar charts showing the number of colonies from **A**, in parental, sgCTRL, and sgKDM6B (sgRNA#1 and sgRNA#2) A-673 cells. **C,** Tumor growth curve of the average volume of xenografts derived from the subcutaneous injection of KDM6B KO cells (sgRNA#1 and sgRNA#2, *n* = 11 each) compared with sgCTRL A-673 cells (*n* = 8, where *n* indicates the number of tumors) in nude athymic mice. **D,** Kaplan–Meier curve representing the percentage of xenograft tumors that reach a tumor volume of 1,000 mm^3^ within days after injection in sgCTRL and KDM6B KO (sgRNA#1 and sgRNA#2) A-673 cells. **E,** IHC staining of CD99, KDM6B, and Ki-67 on sections of tumors excised from sgCTRL and sgKDM6B (sgRNA#1 and sgRNA#2) xenografts of A-673 cells. Black scale bar, 50 µm. H&E, hematoxylin and eosin. **F,** Left,depletion of KDM6A, enriched at single GGAA regions bound by EWSR1::FLI1, perturbs BRG1 recruitment causing repression of target genes in a demethylase-independent manner. KDM6B colocalizes with EWSR1::FLI1 and BRG1 at GGAA repeats, and its depletion results in repression and a mild accumulation of H3K27me3. Statistical significance was determined by the Kruskal–Wallis test with Dunn multiple comparison correction (**B**) and repeated measures two-way ANOVA (**C**). Survival analysis was performed using the log-rank (Mantel–Cox) test (**D**) relative to sgCTRL. Error bars, SEM (**B**). *, *P* < 0.05; **, *P* < 0.01; ***, *P* < 0.001; **F,** Created in BioRender. Figuerola, E. (2025) https://BioRender.com/ikv5h9i.

As KDM6B KO reduces the expression of important targets for Ewing sarcoma tumorigenesis, we tested whether its depletion interferes with tumor growth. Upon KDM6B KO, we observed a lower clonogenic capacity of A-673 cells, supporting a potential role of the demethylase in tumorigenesis (Fig. 7A and B). To assess the relevance of KDM6B *in vivo*, we generated xenografts by subcutaneously injecting sgCTRL, sgKDM6B#1, and sgKDM6B#2 A-673 cells into athymic nude mice. KDM6B KO cells had slower tumor growth and an increase in survival from 18.5 days in the control group to 30 and 33 days in sgKDM6B#1 and sgKDM6B#2, respectively (Fig. 7C and D). Furthermore, IHC analysis of tumors confirmed the expression of the Ewing sarcoma marker CD99 and reduced levels of KDM6B and Ki-67 (Fig. 7E). All these data confirm the role of KDM6B in the maintenance of Ewing sarcoma tumorigenesis.

## Discussion

In this study, we performed an extensive genome-wide analysis of H3K27me3 redistribution upon EWSR1::FLI1 overexpression. Although the maintenance of the overall levels of H3K27me3 was previously described upon EWSR1::FLI1 knockdown (17), published data on H3K27me3 and our results on KDM6 demethylases confirm that locus-specific H3K27me3 changes are relevant to the tumorigenic process (39). Our findings indicate a genome-wide gain of H3K27me3 in relevant genes from EWSR1::FLI1 pathways, supporting the important role of EZH2 in Ewing sarcoma tumorigenesis (9, 38). Nevertheless, the lack of intersection between EZH2 and the oncogene, as already proposed by other groups (16), suggests an indirect repressor role for this Polycomb subunit. Importantly, we have previously described that EWSR1::FLI1 targets weak Polycomb regions in hMSC (20). In this study, we confirmed a set of regions in hpMSC highly enriched in H3K27me3 before oncogene expression, in which the loss of H3K27me3 is concomitant with EWSR1::FLI1 binding, supporting the need for a demethylase activity. Altogether, our results refine the understanding of the early steps of Ewing sarcoma tumorigenesis and the role of the H3K27me3 mark in defining its transformed epigenome.

We demonstrated that KDM6A and KDM6B colocalize with EWSR1::FLI1, shaping two different classes of enhancers that contribute distinctively to the oncogenic process: KDM6A enhancers, enriched in single instances of the GGAA motif, and KDM6B-only enhancers, which are characterized by multimeric GGAA repeats (Fig. 7F). Although GGAA single motifs were described as EWSR1::FLI1-repressed elements, they retain low levels of H3K27ac and transcription (16), aligning with our observation that KDM6A is linked to primed enhancers. These types of enhancers constitute an intermediate state between active and repressed enhancers (52, 53). Indeed, in our study, KDM6A-associated genes containing GGAA single motifs are less transcribed than those enriched with KDM6B. Although these results might suggest an important role of KDM6A in determining cell identity, further analysis of the nature and implications of these primed enhancers and their differential regulation is needed.

It has been shown that the participation of KDM6A in cancer progression is not restricted to its enzymatic activity but to a demethylase-independent function (35, 60). Main changes in H3K27me3 upon depletion of KDM6 demethylases have been described by other groups in regions already containing this repressive mark (26, 30, 61, 62). The maintenance of H3K27me3 in the group of sgCTRL+/sgKDM6A+ peaks, which is constituted by the most intense peaks, reinforces the KDM6A demethylase-independent role. In contrast, the decrease of H3K27me3 observed in the sgCTRL+/sgKDM6B+ group upon KDM6B KO might be due to reduced EZH2 expression. Consistently, the expected H3K27me3 recovery upon KDM6B inhibition is also counterbalanced by the decrease in EZH2 expression in neuroblastoma (61). These results suggest that Ewing sarcoma mechanistically resembles pediatric cancers like neuroblastoma, in which both KDM6B and EZH2 are overexpressed and constitute druggable targets (61, 63). The new set of peaks for both KDM6A and KDM6B KO (sgCTRL−/sgKDM6A+ and sgCTRL−/sgKDM6B+) are not associated with changes in the TSS of KDM6-activated targets, in agreement with a demethylase impact at distal regulatory regions (61). Besides, we observed a mild increase in H3K27me3 levels at specific EWSR1::FLI1 enhancers following KDM6B KO, consistent with the downregulation of these targets upon treatment with the KDM6A/KDM6B demethylase inhibitor GSKJ4. Our results suggest that the effects of GSK-J4, described *in vitro* and *in vivo* by Heisey and colleagues (64), might be due to the specific targeting of the demethylase activity of KDM6B. Indeed, GSK-J4 was effective at the preclinical level for pediatric cancers like neuroblastoma and diffuse intrinsic pontine glioma (65, 66). Although, in hpMSCs, overexpression of EWSR1::FLI1 remodels the chromatin landscape by redistributing H3K27me3, in Ewing sarcoma cell lines, depletion of KDM6B—but not KDM6A—exerts its main effects partly by restoring H3K27me3 levels at enhancers.

We observed KDM6A and KDM6B loss at EWSR1::FLI1-bound regions upon oncogene knockdown, supporting that EWSR1::FLI1 is the main responsible factor for KDM6 recruitment. Interestingly, we found a strong overlap between KDM6A and KDM6B with BRG1 at GGAA single motifs and repeats, respectively. Although the interplay between KDM6B and the BAF complex at GGAA repeats remains to be elucidated, both might work in cooperation to support the strong transcriptional activation orchestrated at GGAA repeats. The recruitment of BRG1 was described at cardiac-specific enhancers as a key step in the activation of cardiac developmental programs (27). Our data reveal that KDM6A recruits BRG1 at EWSR1::FLI1 enhancers in a demethylase-independent manner, with critical consequences for transcriptional activation (Fig. 7F). Given that KDM6A is associated with the H3K4 methyltransferase MLL3/4 complex, a reasonable possibility is that both might regulate gene expression through coordinated histone modifications at primed enhancers (28).

We demonstrated that KDM6A and KDM6B maintain the oncogenic process set by EWSR1::FLI1. Although we defined 260 and 49 EWSR1::FLI1-KDM6A and KDM6B direct targets, respectively, affecting oncogene reprogramming, the contribution of the two demethylases to tumor growth in an oncogene-independent manner should not be dismissed. The role of KDM6A in cancer development is cell-context specific, acting either as a tumor suppressor or as an oncogenic factor (34–37, 67). Ewing sarcoma ranks 12th among cancer malignancies with the highest mRNA expression levels of KDM6A (50). In contrast to the dual role of KDM6A in cancer, KDM6B has been consistently associated with cancer progression (33, 61, 68). Although it is reasonable to speculate that the demethylase function required at early stages of transformation is mainly sustained by KDM6B, further experiments would be needed to determine whether H3K27me3 loss at these early stages is exclusively dependent on KDM6B and whether demethylase-independent mechanisms—particularly involving KDM6A—may also contribute to initial transformation. The presence of KDM6A and KDM6B demethylases at GGAA single and repeat motifs in transformed cells, along with the detrimental effect of their KO on the transcriptional activation of EWSR1::FLI1 targets—concomitant with a reduction in tumor volume—supports their needed role at these positions for maintaining an active transcriptional state.

## Supplementary Material

Supplementary Table S1 Excel file with reagents information.

Supplementary Table S2 Excel file showing genes with gain or loss of H3K27me3 upon EWSR1::FLI1 introduction in hpMSC.

Supplementary Table S3 Excel file showing KDM6A, KDM6B and EWSR1::FLI1 ChIP-seq data.

Supplementary Table S4 Excel file showing associated genes for KDM6A, KDM6B and EWSR1::FLI1 ChIP-seq data.

Supplementary Table S5 Excel file showing DEG and downregulated direct targets upon KDM6A or KDM6B knockdown.

Supplementary Table S6 Excel file showing DEG and downregulated direct targets upon KDM6A or KDM6B knockout.

Supplementary Figure S1 H3K27me3 genome-wide redistribution upon EWSR1::FLI1 overexpression in hpMSCs

Supplementary Figure S2 KDM6A and KDM6B co-localize genome-wide with EWSR1::FLI1 at primed and active enhancers.

Supplementary Figure S3 Knockdown of KDM6A and KDM6B downregulates EWSR1::FLI1-activated targets.

Supplementary Figure S4 KDM6A recruits BRG1 to EWSR1::FLI1-activated enhancers in a demethylase independent manner.

Supplementary Figure S5 KDM6A knockout decreases EwS tumor growth.

Supplementary Figure S6 Impact of KDM6B knockout in EWSR1::FLI1 targets.

## Data Availability

The data generated in this study are publicly available in the Gene Expression Omnibus (GEO) at GSE211743. The ChIP-seq data analyzed in this study (H3K27me3, H3K27ac, and EWSR1::FLI1 in hpMSC and histone marks in A673) were obtained from the GEO at accession numbers GSE106925 and GSE61953. The A673 and EWSR1::ETS-knockdown RNA-seq data analyzed in this study were obtained from the GEO at GSE61953 and GSE176190. The expression array data, including 184 Ewing sarcoma tumors at diagnosis analyzed in this study, were obtained from the GEO at GSE17679, GSE34620, and GSE37371. All other raw data generated in this study are available from the corresponding author upon request.
