# Supplementary material for: KDM6 Demethylases Contribute to EWSR1::FLI1-Driven Oncogenic Reprogramming in Ewing Sarcoma
Source: Cancer Res. 2025 Oct 14;85(22):4485–503. doi: 10.1158/0008-5472.CAN-24-3452 (PMC12616242; doi:10.1158/0008-5472.CAN-24-3452)
Supplement: Supplementary Figure S1 — H3K27me3 genome-wide redistribution upon EWSR1::FLI1 overexpression in hpMSCs [file can-24-3452_supplementary_figure_s1_suppsf1.pdf]

Supplementary Figure 1

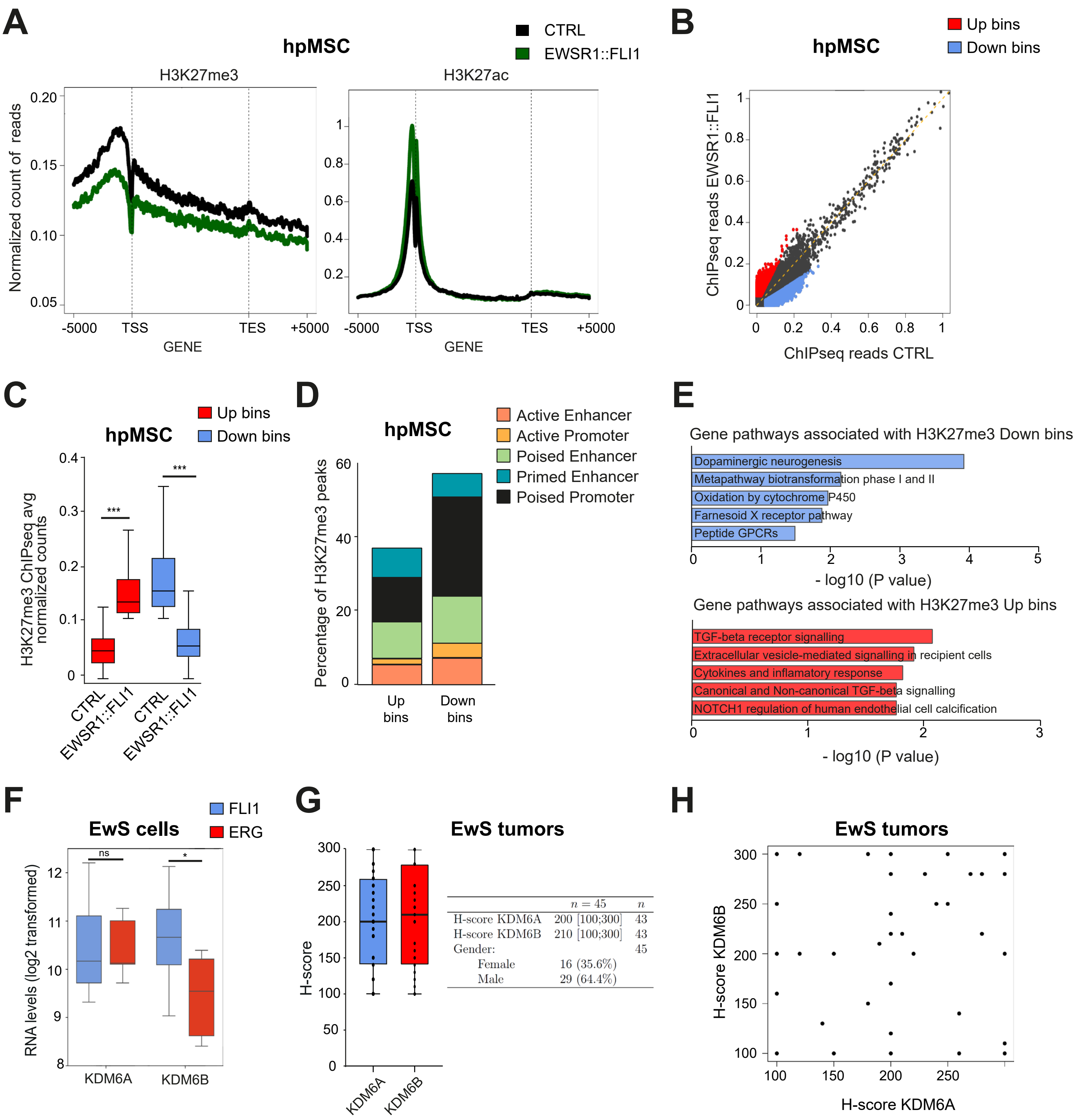

Figure S1. H3K27me3 genome-wide redistribution upon EWSR1::FLI1 overexpression in hpMSCs. (A) Metagene plot showing H3K27me3 (left) and H3K27ac (right) ChIP-seq signals in 6,150 and 15,810 target regions, respectively, at transcription start site (TSS) and transcription end sites (TES) within 5,000 kb window in control (CTRL) and upon infection with the oncogene (EWSR1::FLI1) in hpMSC. (B) Scatterplot of H3K27me3 signal in 3,069,655 bins of 1 kb in CTRL (x-axis) and EWSR1::FLI1 (y-axis) hpMSC (R2=0.582, slope=0.703). Bins that significantly gain (Up bins) or lose H3K27me3 (Down bins) are highlighted in red or blue, respectively. (C) Boxplot depicting the average ChIP-seq signal of H3K27me3 in Up and Down bins in CTRL and EWSR1::FLI1 hpMSC. (D) Bar plot depicting percentage of annotated regulatory elements (active/poised/primed enhancers and active/poised promoters) covered by at least one bin of the genome (1 kb) in Up bins or Down bins of H3K27me3 in CTRL and EWSR1::FLI1 hpMSC. (E) Bar chart representing the top five enriched signaling pathways of 2,879 genes from H3K27me3 Down bins (above) and 2,621 genes from Up bins (below) from CTRL and EWSR1::FLI1 hpMSC and their associated P-value. (F) Boxplot representing the RNA levels of KDM6A and KDM6B in EwS cell lines containing FLI1 or ERG EWSR1::ETS fusion from Orth *et al*. (45). (G) Boxplot depicting immunohistochemical score (H-score) mean values for KDM6A and KDM6B (left) in our cohort of 45 EwS primary tumors (right). Individual H-score values are represented as dots. Table shows descriptive statistics information of the recruited samples (right). (H) Scatter plot of individual H-score values in EwS primary tumors for KDM6A (x-axis) and KDM6B (y-axis) (r=0.000, CI=(-0.308,0.308), P-value=1.00). Statistical differences between groups were assessed by Wilcoxon signed-rank test (C) and Student t-test (F). Error bars indicate SD (C), (F), and (G). P***<0.001, *P<0.05 and ns indicates not significant.
