# Supplementary material for: KDM6 Demethylases Contribute to EWSR1::FLI1-Driven Oncogenic Reprogramming in Ewing Sarcoma
Source: Cancer Res. 2025 Oct 14;85(22):4485–503. doi: 10.1158/0008-5472.CAN-24-3452 (PMC12616242; doi:10.1158/0008-5472.CAN-24-3452)
Supplement: Supplementary Figure S2 — KDM6A and KDM6B co-localize genome-wide with EWSR1::FLI1 at primed and active enhancers. [file can-24-3452_supplementary_figure_s2_suppsf2.pdf]

Supplementary Figure 2

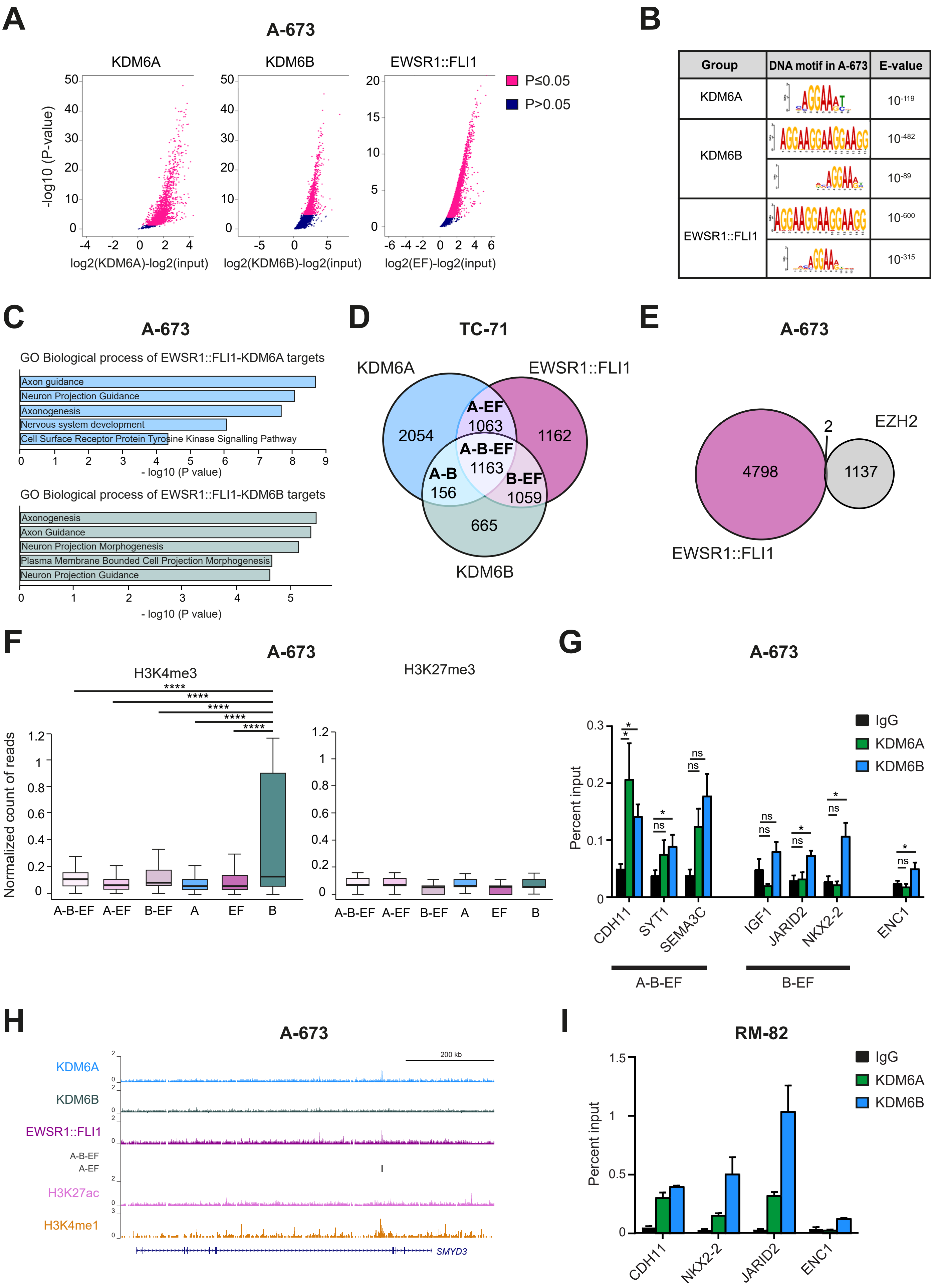

Figure S2. KDM6A and KDM6B co-localize genome-wide with EWSR1::FLI1 at primed and active enhancers. (A) Volcano plots of the significant peaks identified by DiffBind for KDM6A, KDM6B, and EWSR1::FLI1 (3,737, 2,687, and 4,800 respectively) (P value ≤ 0.05 for KDM6A and EWSR1::FLI1, and FDR < 10^{-5} for KDM6B) in A-673 cells. (B) Table showing the top MEME DNA motifs enriched for KDM6A, KDM6B and EWSR1::FLI1 peaks and the corresponding E-value for every set of peaks. (C) Bar chart representing the top five enriched gene ontology (GO) biological processes of the 921 or 893 genes associated to EWSR1::FLI1 and KDM6A (above) or KDM6B peaks (below), respectively, and their associated P-value. (D) Venn diagram showing overlap of KDM6A, KDM6B, and EWSR1::FLI1 at peak level in TC-71 cells. (E) Same as (D) showing overlap between EWSR1::FLI1 and EZH2 at peak level in A-673 cells. (F) Boxplot depicting the average ChIP-seq signal of H3K4me3 (left) and H3K27me3 (right) in each set of peaks in A-673 cells. (G) ChIP-qPCR of KDM6A and KDM6B in a set of EWSR1::FLI1-bound regions with both KDM6A and KDM6B (A-B-EF) or only KDM6B (B-EF) in A-673 cells. (H) UCSC genome browser signal tracks for KDM6A, KDM6B, EWSR1::FLI1, H3K27ac, and H3K4me1 at the *SMYD3* gene in A-673. EWSR1::FLI1 and KDM6A peaks with or without KDM6B (A-B-EF or A-EF, respectively) are represented as black bars below tracks. (I) ChIP-qPCR of KDM6A and KDM6B in RM-82 cells containing the EWSR1::ERG-fusion. For (G) and (I) *ENC1* was used as a negative control region. Statistical significance between groups was assessed by Wilcoxon signed-rank test (F), Kruskal-Wallis test with Dunn's correction for multiple comparisons (G) and Mann-Whitney t-test (I). Error bars indicate SD (F) or SEM of three independent biological experiments (G) and (I). ****P<0.0001, *P<0.05 and ns indicates not significant.
