# Supplementary material for: KDM6 Demethylases Contribute to EWSR1::FLI1-Driven Oncogenic Reprogramming in Ewing Sarcoma
Source: Cancer Res. 2025 Oct 14;85(22):4485–503. doi: 10.1158/0008-5472.CAN-24-3452 (PMC12616242; doi:10.1158/0008-5472.CAN-24-3452)
Supplement: Supplementary Figure S3 — Knockdown of KDM6A and KDM6B downregulates EWSR1::FLI1-activated targets. [file can-24-3452_supplementary_figure_s3_suppsf3.pdf]

Supplementary Figure 3

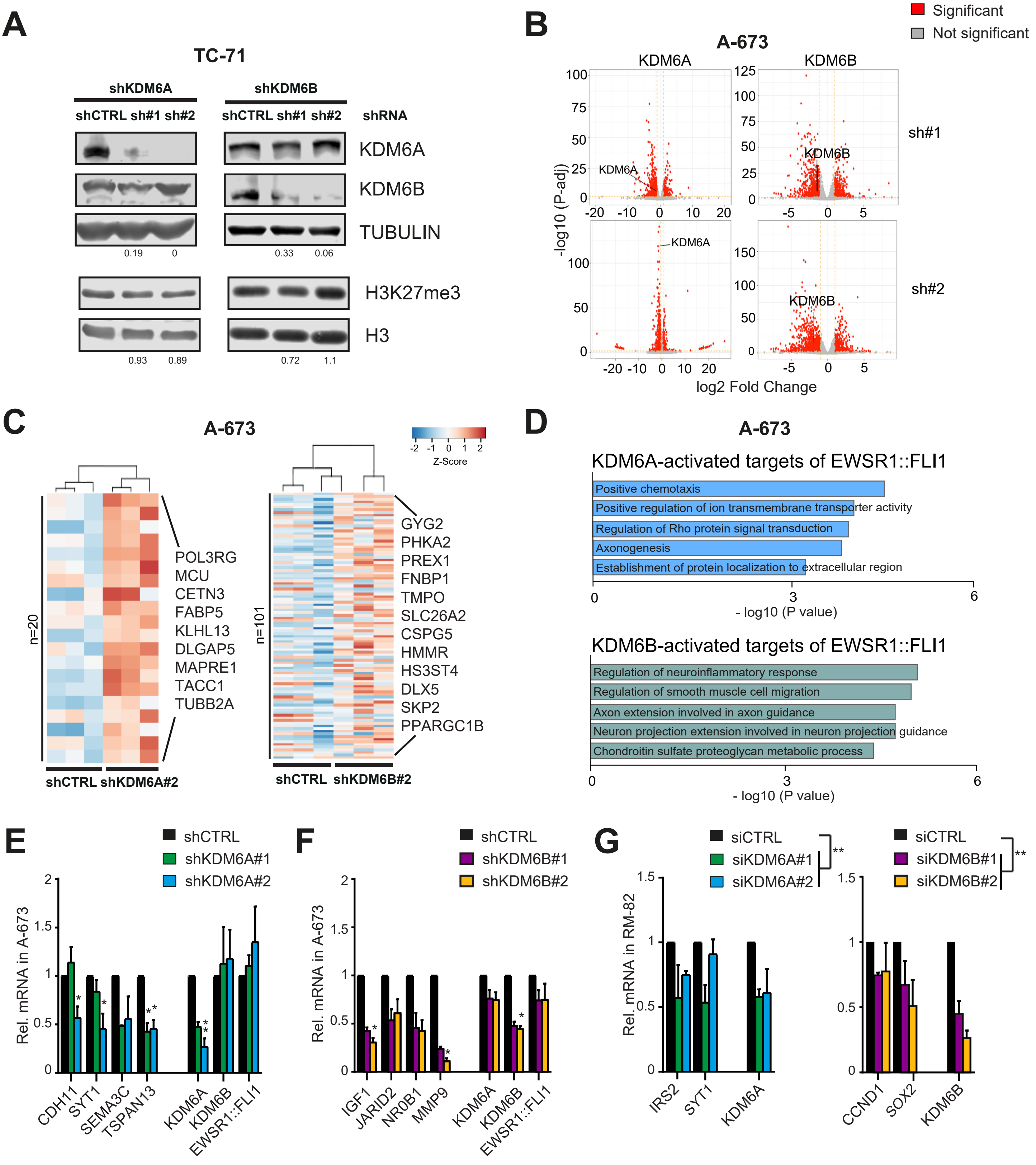

Figure S3. Knockdown of KDM6A and KDM6B downregulates EWSR1::FLI1-activated targets. (A) Western blot showing levels of KDM6A and KDM6B in whole cell (above) or H3K27me3 in histone extracts (below) upon KDM6A or KDM6B knockdown with two doxycycline-inducible shRNA sequences (sh#1 and sh#2) at 72 hours in TC-71 cells. Tubulin and histone H3 were used as loading controls. Numbers below represent band intensity quantification of KDM6A, KDM6B or H3K27me3 normalized to loading control and relative to shCTRL. (B) Volcano plots depicting log fold change (x-axis) and the associated log P-adjusted value (y-axis) of deregulated targets upon KDM6A (left) or KDM6B (right) knockdown with #sh1 or #sh2 sequences (above or below, respectively). Statistically significant deregulated targets according to set cut-off are highlighted in red. (C) Heatmap showing expression levels of genes in the vicinity of EWSR1::FLI1-KDM6A and EWSR1::FLI1-KDM6B ChIP-seq peaks (100 kb) that are significantly upregulated upon knockdown of each demethylase in A-673 cells. n, indicates number of deregulated direct targets upon knockdown in each panel. (D) Bar chart representing the top five enriched gene ontology (GO) biological processes and their associated P-value of the 64 genes in the vicinity (100 kb) of EWSR1::FLI1-KDM6A peaks (above) and the 139 genes in the vicinity (100 kb) of EWSR1::FLI1-KDM6B peaks (below) that are significantly downregulated upon knockdown of each demethylase in A-673. (E) RT-qPCR determination of mRNA expression of EWSR1::FLI1 targets with active enhancers in shCTRL and shKDM6A (#1 and 2) in A-673 cells. Values were normalized to *GAPDH* and relative to shCTRL. (F) Same analysis as in (E) for shCTRL and shKDM6B (#1 and 2) in A-673 cells. (G) RT-qPCR determination of mRNA expression levels of EWSR1::FLI1 targets with active enhancers in siCTRL and siKDM6A (#1 and 2) (left) and siKDM6B (#1 and 2) (right) in RM-82 cells. Values were normalized to *TBP* and relative to siCTRL. Statistical significance was determined by Kruskal-Wallis test with Dunn's multiple comparison correction related to sgCTRL (E) and (F), and Mann-Whitney t-test of the two siRNA sequences compared to control (G). Error bars indicate SEM (E) and (F) and SD (G). $^{**}P < 0.01$, and $^{*}P < 0.05$.
