# Supplementary material for: KDM6 Demethylases Contribute to EWSR1::FLI1-Driven Oncogenic Reprogramming in Ewing Sarcoma
Source: Cancer Res. 2025 Oct 14;85(22):4485–503. doi: 10.1158/0008-5472.CAN-24-3452 (PMC12616242; doi:10.1158/0008-5472.CAN-24-3452)
Supplement: Supplementary Figure S4 — KDM6A recruits BRG1 to EWSR1::FLI1-activated enhancers in a demethylase independent manner. [file can-24-3452_supplementary_figure_s4_suppsf4.pdf]

Supplementary Figure 4

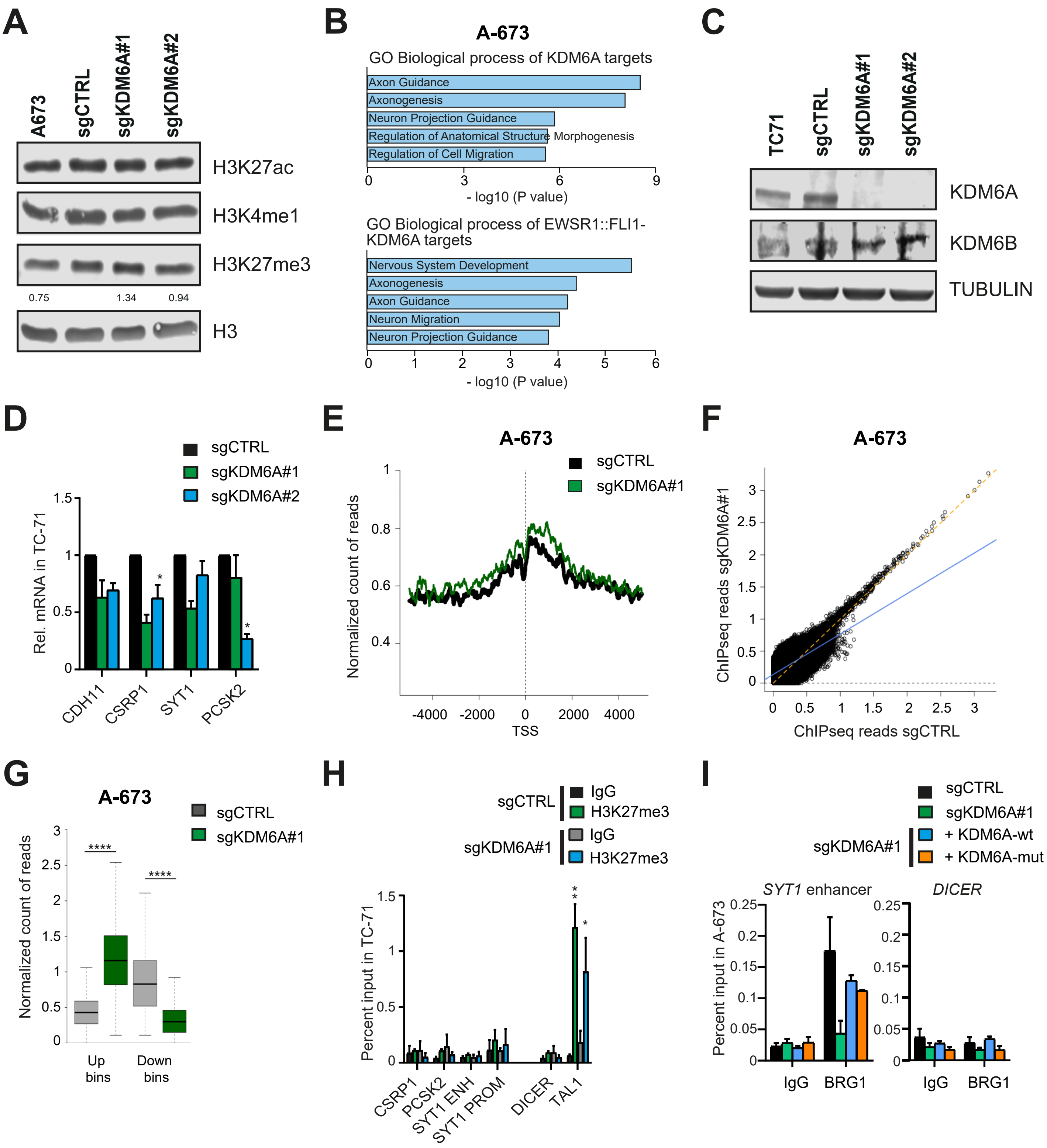

Figure S4. KDM6A recruits BRG1 to EWSR1::FLI1-activated enhancers in a demethylase-independent manner. (A) Western blot showing levels of H3K27ac, H3K4me1, and H3K27me3 in histone extracts upon KDM6A KO with two sgRNA sequences (#1 and #2) in A-673 cells. Histone H3 was used as loading control. Numbers below represent band quantification of H3K27me3 normalized to H3 and relative to non-targeting control (sgCTRL). (B) Bar chart representing the top five enriched gene ontology (GO) biological processes and their associated P-value of the set of differentially expressed genes for KDM6A KO (above) and for EWSR1::FLI1-KDM6A direct targets (below). (C) Western blot showing levels of KDM6A, and KDM6B in whole cell extracts upon KDM6A KO with two sgRNA sequences (#1 and #2) in TC-71 cells. Tubulin was used as loading control. (D) RT-qPCR determination of EWSR1::FLI1 targets with both KDM6A and KDM6B ChIP-seq peaks (A-B-EF group) in TC-71 KO cells (sgRNA #1 and #2). *GAPDH* was used as housekeeping gene. (E) Metagene plot showing H3K27me3 ChIP-seq signal of 1,245 KDM6A-activated targets from RNA-seq data at transcription start site (TSS) within 5,000 kb window in sgCTRL and sgKDM6A#1 in A-673 cells. (F) Scatter plot of H3K27me3 ChIP-seq signal in 3,095,665 bins of 1 kb in sgCTRL (x-axis) and sgKDM6A#1 (y-axis) (R^2=0.326, slope=0.631). (G) Boxplot depicting the average ChIP-seq signal of H3K27me3 in 1 kb bins in sgCTRL and sgKDM6A#1. Bin mapping analysis identified 579,992 and 142,556 bins that gained (Up bins) or loss (Down bins) H3K27me3 signal, respectively, upon KDM6A KO compared to control. (H) ChIP-qPCR of H3K27me3 enrichment in the enhancer region of *CSRP1*, *PCSK2* and *SYT1* (ENH) and in *SYT1* promoter (PROM) region upon KDM6A KO sgRNA#1 in TC-71 cells. *DICER* and *TAL1* were used as negative and positive control regions, respectively. (I) ChIP-qPCR of BRG1 enrichment at the enhancer region of *SYT1* KDM6A-activated target in sgCTRL+empty vector (sgCTRL), sgKDM6A#1+empty vector (sgKDM6A#1), and upon overexpression of KDM6A wild-type (sgKDM6A#1+KDM6A-wt) and the dead mutant (H1146A/E1148A; sgKDM6A#1+KDM6A-mut) forms in sgKDM6A#1. *DICER* was used as a negative control region. Statistical significance was determined by Kruskal-Wallis test with Dunn's multiple comparison correction related to sgCTRL (D), Wilcoxon signed-rank test (G), ordinary two way ANOVA test with Holm-Šídák multiple comparisons correction (H) compared to control and Mann-Whitney t-test of each condition compared to sgCTRL (I). Error bars indicate SD (G) and (I) or SEM (D) and (H) of three independent biological experiments. ****P< 0.0001, ***P < 0.01, and *P < 0.05.
