# Supplementary material for: KDM6 Demethylases Contribute to EWSR1::FLI1-Driven Oncogenic Reprogramming in Ewing Sarcoma
Source: Cancer Res. 2025 Oct 14;85(22):4485–503. doi: 10.1158/0008-5472.CAN-24-3452 (PMC12616242; doi:10.1158/0008-5472.CAN-24-3452)
Supplement: Supplementary Figure S5 — KDM6A knockout decreases EwS tumor growth. [file can-24-3452_supplementary_figure_s5_suppsf5.pdf]

Supplementary Figure 5

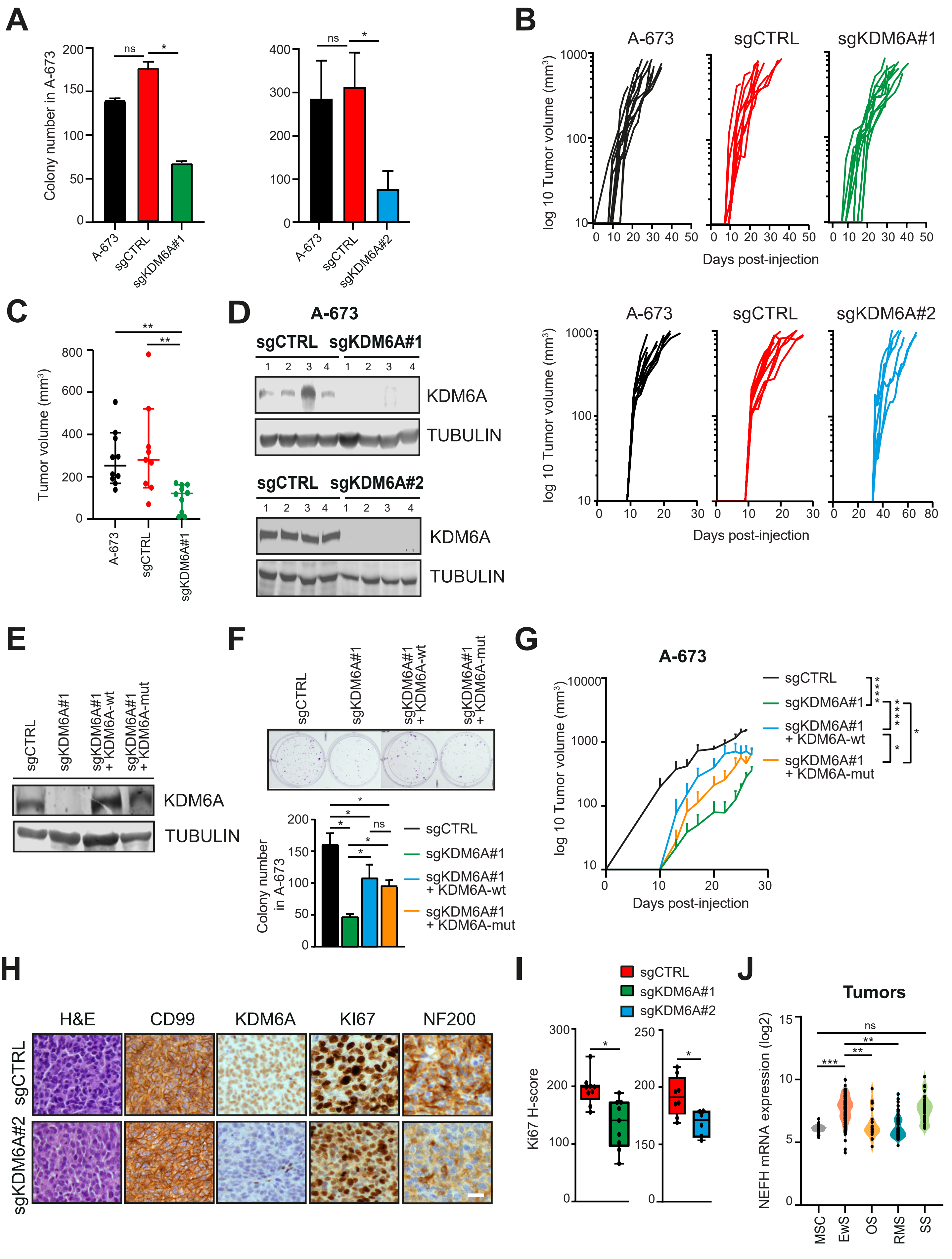

Figure S5. KDM6A knockout decreases EwS tumor growth. (A) Bar charts showing number of colonies from Fig. 5A, in parental, sgCTRL and sgKDM6A (sgRNA #1 and 2) A-673 cells. (B) Spaghetti plots showing tumor volume of xenografts from parental, sgCTRL, and sgKDM6A (sgRNA #1 and 2) in A-673 cells. (C) Boxplot representing tumor volume at 17 days post-injection of parental, sgCTRL, and KDM6A KO cells (sgRNA#1) in A-673. Each dot represents an individual tumor volume. (D) Western blot showing protein levels of KDM6A in 8 representative xenograft tumors derived from sgCTRL and 4 from sgKDM6A (sgRNA#1 and 2) in A-673. (E) Western blot of KDM6A levels in control+empty vector (sgCTRL), KDM6A KO cells+empty vector (sgKDM6A#1) and in sgKDM6A#1 overexpressing the wild-type (sgKDM6A#1+KDM6A-wt) or dead mutant (H1146A/E1148A; sgKDM6A#1+KDM6A-mut) forms of KDM6A in A-673 cells. (F) Colony formation assay (above) and bar charts showing number of colonies (below) in sgCTRL, sgKDM6A#1, sgKDM6A#1+KDM6A-wt and sgKDM6A#1+KDM6A-mut in A-673 cells. (G) Tumor growth curves of the average volume of xenografts derived from subcutaneous injection of sgCTRL (n=11), sgKDM6A#1 (n=9), sgKDM6A#1+KDM6A-wt (n=8) and sgKDM6A#1+KDM6A-mut (n=11) in A-673 cells. (H) Immunohistochemistry staining of CD99, KDM6A, Ki67, and NF200 on sections of tumors excised from sgCTRL and sgKDM6A (#2) xenografts of A-673 cells. CD99 was used as positive control for EwS cells, Ki67 for cell proliferation, and hematoxylin-eosin for histopathological evaluation of tissue. White scale bar represents 50 µm. (I) Boxplot depicting H-score quantification of Ki67 in sgCTRL and sgKDM6A (#1 and #2) in immunohistochemistry sections from Fig. 5F and S5H. (J) Violin plot representing mRNA levels of *NEFH* in primary tumors from GEO public data repositories including EwS among other primary sarcoma tumors including osteosarcoma (OS), rhabdomyosarcoma (RMS), and synovial sarcoma (SS). MSCs derived from the healthy bone marrow were included as control cells. For (D) and (E) tubulin was used as loading control. Statistical significance was determined by Kruskal-Wallis test with Dunn's multiple comparison correction (A, C and I) relative to sgCTRL, Mann-Whitney t-test (F), ordinary two-way ANOVA with Dunnet multiple comparisons test compared to sgKDM6A#1 and to sgKDM6A#1+KDM6A-wt (G), and ordinary one-way ANOVA with Holm-Šídák multiple comparison test (J) relative to EwS. Error bars indicate SEM. ****P< 0.0001, ***$P \leq 0.001$, **$P \leq 0.01$, *$P<0.05$, ns indicates not significant.
