# Supplementary material for: KDM6 Demethylases Contribute to EWSR1::FLI1-Driven Oncogenic Reprogramming in Ewing Sarcoma
Source: Cancer Res. 2025 Oct 14;85(22):4485–503. doi: 10.1158/0008-5472.CAN-24-3452 (PMC12616242; doi:10.1158/0008-5472.CAN-24-3452)
Supplement: Supplementary Figure S6 — Impact of KDM6B knockout in EWSR1::FLI1 targets. [file can-24-3452_supplementary_figure_s6_suppsf6.pdf]

Supplementary Figure 6

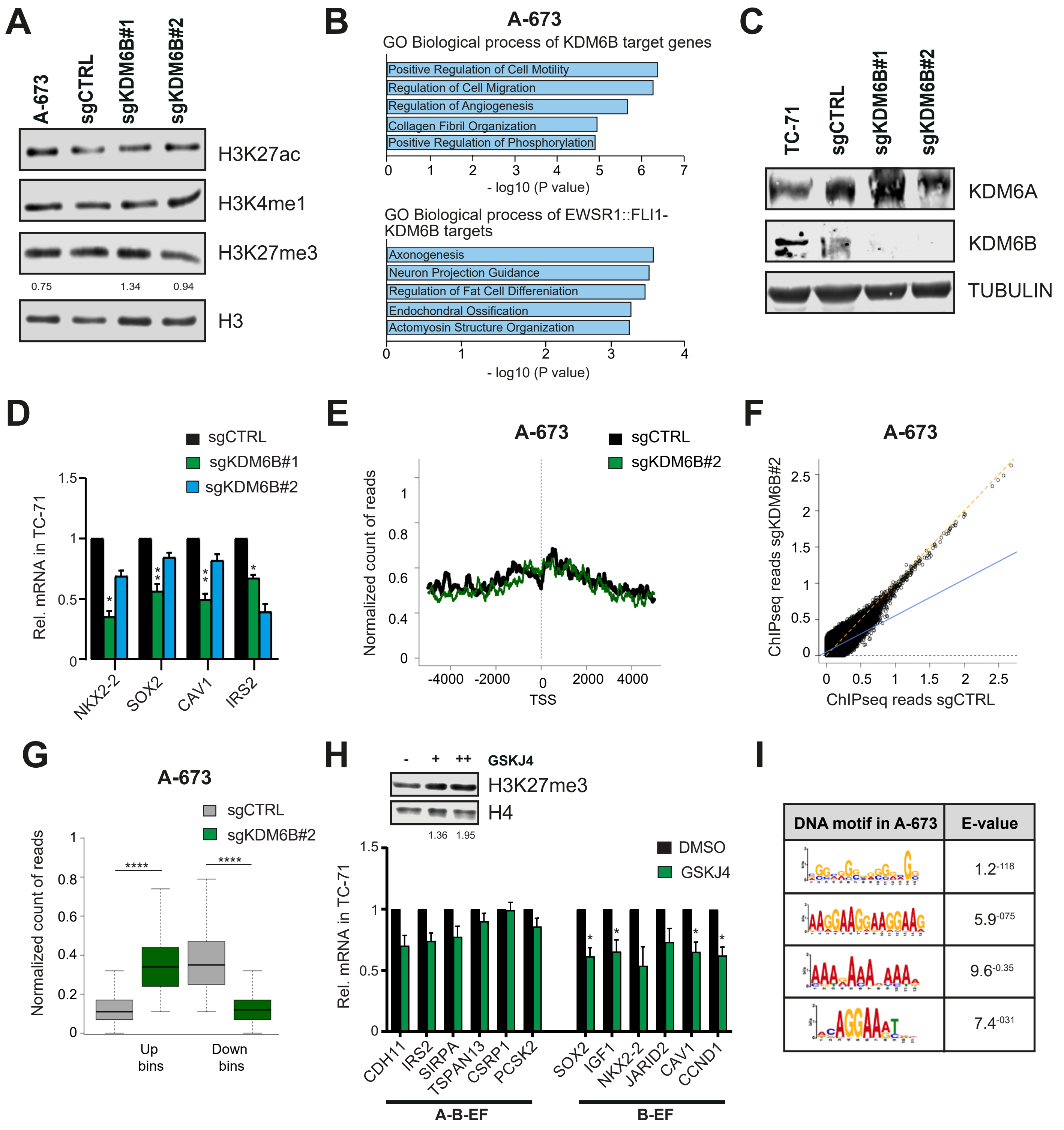

Figure S6. Impact of KDM6B knockout in EWSR1::FLI1 targets. (A) Western blot showing levels of H3K27ac, H3K4me1, and H3K27me3 in histone extracts upon KDM6B KO with two sgRNA sequences (#1 and #2) in A-673 cells. Histone H3 was used as loading control. Numbers below represent band quantification of H3K27me3 normalized to H3 and relative to non-targeting control (sgCTRL). (B) Bar chart representing the top five enriched gene ontology (GO) biological processes and their associated P-value of the set of differentially expressed genes for KDM6B KO (above) and for EWSR1::FLI1-KDM6B direct targets (below). (C) Western blot showing levels of KDM6A, and KDM6B in whole cell extracts upon KDM6B KO with two sgRNA sequences (#1 and #2) in TC-71 cells. Tubulin was used as loading control. (D) RT-qPCR of EWSR1::FLI1-KDM6B targets in sgKDM6B#1 and #2 TC-71 cells. *GAPDH* was used as housekeeping gene. (E) Metagene plot showing H3K27me3 ChIP-seq signal of 266 KDM6B-activated targets from RNA-seq data at transcription start site (TSS) within 5,000 kb window in sgCTRL and sgKDM6B#2 in A-673 cells. (F) Scatter plot of H3K27me3 ChIP-seq signal in 3,095,665 bins of 1 kb in sgCTRL (x-axis) and sgKDM6B#1 (y-axis) (R^2=0.285, slope=0.501). (G) Boxplot depicting the average ChIP-seq signal of H3K27me3 in 1 kb bins in sgCTRL and sgKDM6B#2. Bin mapping analysis identified 380,371 and 442,200 bins that gained (Up bins) or loss (Down bins) H3K27me3 signal, respectively, upon KDM6B KO compared to control. (H) Western blot (above) showing levels of H3K27me3 in histone extracts of TC-71 cells treated with vehicle or the demethylase inhibitor GSKJ4 at 2.5 and 5 µM (+ and ++, respectively) for 72h. Histone H4 was used as loading control. Numbers below represent band quantification of H3K27me3 normalized to H4 and relative to vehicle control. RT-qPCR (below) determination of EWSR1::FLI1 targets with both KDM6A and KDM6B or with KDM6B ChIP-seq peaks (A-B-EF and B-EF groups, respectively) in TC-71 cells treated with vehicle or GSK-J4 at 2.5 µM for 72 hours. *TBP* was used as housekeeping gene. (I) Table showing top MEME DNA motifs and the corresponding E-value for BRG1 ChIP-seq peaks in A-673 cells. Statistical significance was determined by Kruskal-Wallis test with Dunn's correction for multiple comparison (D), Wilcoxon signed-rank test (G) and Student t-test (H) related to control group. Error bars in (D and H) indicate SEM of three independent biological experiments; ****P<0.0001, ***P < 0.01* and *P < 0.05.
